# PRMD: an integrated database for plant RNA modifications

**DOI:** 10.1093/nar/gkad851

**Published:** 2023-10-13

**Authors:** Xiaoqiang Lang, Chunyan Yu, Mengyuan Shen, Lei Gu, Qian Qian, Degui Zhou, Jiantao Tan, Yiliang Li, Xin Peng, Shu Diao, Zhujun Deng, Zhaohui Ruan, Zhi Xu, Junlian Xing, Chen Li, Runfeng Wang, Changjun Ding, Yi Cao, Qi Liu

**Affiliations:** State Key Laboratory of Tree Genetics and Breeding, Key Laboratory of Tree Breeding and Cultivation of State Forestry Administration, Research Institute of Forestry, Chinese Academy of Forestry, Beijing 100091, China; Microbiology and Metabolic Engineering Key Laboratory of Sichuan Province, College of Life Science, Sichuan University, Chengdu, Sichuan, 610041, China; Frontiers Science Center for Disease-related Molecular Network, Laboratory of Omics Technology and Bioinformatics, West China Hospital, Sichuan University, Chengdu, Sichuan, 610041, China; Rice Research Institute, Guangdong Academy of Agricultural Sciences, Key Laboratory of Genetics and Breeding of High Quality Rice in Southern China (Co-construction by Ministry and Province), Ministry of Agriculture and Rural Affairs, Guangdong Key Laboratory of New Technology in Rice Breeding, Guangdong Rice Engineering Laboratory, Guangzhou, 510640, China; Epigenetics Laboratory, Max Planck Institute for Heart and Lung Research & Cardiopulmonary Institute (CPI). Parkstr.1 61231 Bad Nauheim Germany; Rice Research Institute, Guangdong Academy of Agricultural Sciences, Key Laboratory of Genetics and Breeding of High Quality Rice in Southern China (Co-construction by Ministry and Province), Ministry of Agriculture and Rural Affairs, Guangdong Key Laboratory of New Technology in Rice Breeding, Guangdong Rice Engineering Laboratory, Guangzhou, 510640, China; Rice Research Institute, Guangdong Academy of Agricultural Sciences, Key Laboratory of Genetics and Breeding of High Quality Rice in Southern China (Co-construction by Ministry and Province), Ministry of Agriculture and Rural Affairs, Guangdong Key Laboratory of New Technology in Rice Breeding, Guangdong Rice Engineering Laboratory, Guangzhou, 510640, China; Rice Research Institute, Guangdong Academy of Agricultural Sciences, Key Laboratory of Genetics and Breeding of High Quality Rice in Southern China (Co-construction by Ministry and Province), Ministry of Agriculture and Rural Affairs, Guangdong Key Laboratory of New Technology in Rice Breeding, Guangdong Rice Engineering Laboratory, Guangzhou, 510640, China; Guangdong Provincial Key Laboratory of Silviculture, Protection and Utilization/Guangdong Academy of Forestry, Guangzhou, Guangdong 510520, China; Rice Research Institute, Guangdong Academy of Agricultural Sciences, Key Laboratory of Genetics and Breeding of High Quality Rice in Southern China (Co-construction by Ministry and Province), Ministry of Agriculture and Rural Affairs, Guangdong Key Laboratory of New Technology in Rice Breeding, Guangdong Rice Engineering Laboratory, Guangzhou, 510640, China; Research Institute of Subtropical Forestry, Chinese Academy of Forestry, Hangzhou, China; Precision Medicine Center, Precision Medicine Key Laboratory of Sichuan Province, West China Hospital, Sichuan University, Chengdu, Sichuan, 610041, China; Sun Yat-sen University Cancer Center, State Key Laboratory Oncology in South China, Collaborative Innovation Center of Cancer Medicine, 510060, Guangzhou, China; Guangxi Key Laboratory of Images and Graphics Intelligent Processing, Guilin University of Electronics Technology, Guilin, 541004, China; Rice Research Institute, Guangdong Academy of Agricultural Sciences, Key Laboratory of Genetics and Breeding of High Quality Rice in Southern China (Co-construction by Ministry and Province), Ministry of Agriculture and Rural Affairs, Guangdong Key Laboratory of New Technology in Rice Breeding, Guangdong Rice Engineering Laboratory, Guangzhou, 510640, China; Rice Research Institute, Guangdong Academy of Agricultural Sciences, Key Laboratory of Genetics and Breeding of High Quality Rice in Southern China (Co-construction by Ministry and Province), Ministry of Agriculture and Rural Affairs, Guangdong Key Laboratory of New Technology in Rice Breeding, Guangdong Rice Engineering Laboratory, Guangzhou, 510640, China; Guangdong Provincial Key Laboratory of Crop Genetic Improvement, Crops Research Institute, Guangdong Academy of Agricultural Sciences, Guangzhou, China; State Key Laboratory of Tree Genetics and Breeding, Key Laboratory of Tree Breeding and Cultivation of State Forestry Administration, Research Institute of Forestry, Chinese Academy of Forestry, Beijing 100091, China; Microbiology and Metabolic Engineering Key Laboratory of Sichuan Province, College of Life Science, Sichuan University, Chengdu, Sichuan, 610041, China; Rice Research Institute, Guangdong Academy of Agricultural Sciences, Key Laboratory of Genetics and Breeding of High Quality Rice in Southern China (Co-construction by Ministry and Province), Ministry of Agriculture and Rural Affairs, Guangdong Key Laboratory of New Technology in Rice Breeding, Guangdong Rice Engineering Laboratory, Guangzhou, 510640, China

## Abstract

The scope and function of RNA modifications in model plant systems have been extensively studied, resulting in the identification of an increasing number of novel RNA modifications in recent years. Researchers have gradually revealed that RNA modifications, especially N^6^-methyladenosine (m^6^A), which is one of the most abundant and commonly studied RNA modifications in plants, have important roles in physiological and pathological processes. These modifications alter the structure of RNA, which affects its molecular complementarity and binding to specific proteins, thereby resulting in various of physiological effects. The increasing interest in plant RNA modifications has necessitated research into RNA modifications and associated datasets. However, there is a lack of a convenient and integrated database with comprehensive annotations and intuitive visualization of plant RNA modifications. Here, we developed the Plant RNA Modification Database (PRMD; http://bioinformatics.sc.cn/PRMD and http://rnainformatics.org.cn/PRMD) to facilitate RNA modification research. This database contains information regarding 20 plant species and provides an intuitive interface for displaying information. Moreover, PRMD offers multiple tools, including RMlevelDiff, RMplantVar, RNAmodNet and Blast (for functional analyses), and mRNAbrowse, RNAlollipop, JBrowse and Integrative Genomics Viewer (for displaying data). Furthermore, PRMD is freely available, making it useful for the rapid development and promotion of research on plant RNA modifications.

## Graphical Abstract

**Graphical Abstract:**
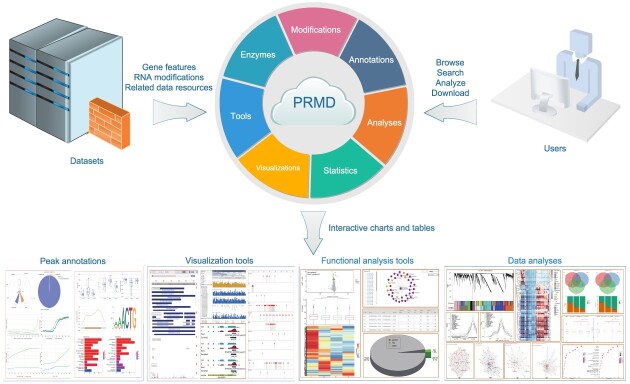

## Introduction

RNA modifications provide diverse RNA molecules with an additional layer of information during epitranscriptome-mediated post-transcriptional regulation (1). More than 160 types of RNA modifications have been identified, including N^1^-methyladenosine (m^1^A), C^5^-methylcytosine (m^5^C), N^6^-methyladenosine (m^6^A), N^7^-methylguanosine (m^7^G), N^4^-acetylcytidine (ac^4^C), 2′-O-Me and pseudouridine, which can regulate the RNA secondary structure, expression, splicing, stability and translation (2–4). Specifically, m^6^A, which is one of the most prevalent internal modifications of diverse RNA molecules, has been detected in messenger RNAs (mRNAs) and accounts for approximately 50% of the methylated nucleotides in RNA (5–7). Numerous studies have shown that RNA modifications are important for gene regulation and affect various aspects of plant development, stress responses and evolution (8,9). For example, the inactivation of a key *Arabidopsis thaliana* m^6^A methyltransferase (mRNA adenosine methylase) can lead to embryo lethality (10). In rice, a lack of the m^5^C methyltransferase OsNSUN2 increases susceptibility to heat stress (11). The evolution of m^6^A is at least in part related to genome replication events in complex polyploid plant genomes (12).

To date, many studies have developed new sequencing methods to identify different RNA modifications (e.g. m^6^A-seq, m^5^C-RIP-seq and m^1^A-seq) (13,14). For example, the methylated RNA immunoprecipitation sequencing (MeRIP-seq) analysis, which combines immunoprecipitation and next-generation sequencing, has been widely used to quantitatively explore RNA modifications throughout the transcriptome. Using these methods, substantial amounts of sequencing data have been obtained for a variety of species, including mammals, yeast and plants. Several databases and web servers, such as CVm6A (15), SRAMP (16) and WHISTLE (17), have been constructed to integrate the existing sequencing data for mammals. In contrast, there are only a few web servers, such as AthMethPre (18) and RFAthM6A (19), for predicted RNA modifications in plants. There are other databases that include RNA modifications from a few plant species. For example, RMBase v2.0 (20), MeT-DB v2.0 (21) and REPIC (22) contain information for only one plant species (*A. thaliana*), whereas m6A-Atlas 2.0 (23) and ENCORE (https://rna.sysu.edu.cn/encore/) provide details regarding 6 and 10 plant species, respectively. Additionally, RNAmod (24) consists of annotated RNA modifications from 21 species, but only a few of these species are plants (24). Both PEA, which is an integrated R toolkit for plant epitranscriptome analyses, and its updated version deepEA, which is a containerized web-based platform for interactive analyses of epitranscriptome sequencing data, focus exclusively on m^6^A peak analyses and annotations, with no information regarding other RNA modifications (25,26). Because of the considerable accumulation of datasets for RNA modifications and related annotation datasets in different plant tissues at various developmental stages and under diverse stress conditions, there is an urgent need for a comprehensive database that integrates these large-scale datasets and their extensive annotations.

We herein present the Plant RNA Modification Database (PRMD), a comprehensive plant RNA modification database that integrates the RNA modifications from up to 20 diverse plant species. As well as the m^6^A modification, we collected other diverse known RNA modifications (m^1^A, m^5^C, m^7^G, ac^4^C, 2′-O-Me and pseudouridine) and related datasets (e.g. RNA secondary structures and RBP binding sites) from published research articles and related plant resources. Additionally, PRMD provides various useful tools for functional analyses and data visualization, such as RMlevelDiff, RMplantVar, RNAmodNet and Blast (for data analyses) as well as mRNAbrowse, RNAlollipop, JBrowse and Integrative Genomics Viewer (IGV) (for intuitively visualizing the data in PRMD). Using PRMD, we determined that RNA modifications are common in plants. Moreover, the genes encoding transcripts with the m^6^A modification were under purifying selection during evolution. Details regarding the significant effects of m^6^A modifications on alternative polyadenylation (APA), exon usage, gene expression and translational efficiency (TE) are available in PRMD. In summary, PRMD is an integrated and intuitive database developed specifically for research on RNA modifications in plants, making it relevant for the rapidly developing plant epitranscriptomics-related research fields.

## Materials and methods

### Data sources and reference genomes

Reference genome sequences and gene annotation files of plant species were obtained from the EnsemblPlants database (27), with the exception of the reference genomes of five species, namely *Gossypium arboretum, Gossypium hirsutum, Fragaria vesca, Nicotiana benthamiana* and *Paulownia fortunei*, which were retrieved from the Cotton Functional Genomics Database (CottonFGD) (28), Genome Database for Rosaceae (GDR) (29), Phytozome (30), Solanaceae Genomics Network (SGN) (31) and National Center for Biotechnology Information (NCBI: https://www.ncbi.nlm.nih.gov/), respectively. For the species that have only GFF3 format gene annotation files, the Gffread program (32) was used to convert the GFF format to the GTF format, which was used for annotating RNA modification. The methylated RNA immunoprecipitation sequencing (MeRIP-seq) datasets were collected from the Sequence Read Archive (SRA) database of NCBI (https://www.ncbi.nlm.nih.gov/sra) and the Genome Sequence Archive (GSA) database (33) of the National Genomics Data Center (NGDC) (34) (Supplementary Table S1).

Other types of known RNA modifications, such as m^1^A, m^5^C, m^7^G, ac^4^C, 2′-O-Me and pseudouridine, were collected from published research articles and other RNA modification resources (Supplementary Tables S2 and S3). The modification sites in nanopore sequencing datasets were obtained from the DirectRMDB database (35), whereas the nucleotide-resolution modification sites in miCLIP datasets were obtained from the m^6^A-Altas2.0 database. We also integrated additional related datasets, including expression quantitative trait locus (eQTL) datasets from the AtMAD (36) and Rice-eQTL (http://riceqtl.ncpgr.cn/) databases, genome-wide association study (GWAS) datasets from the GWAS Atlas database (37), RNA G-quadruplex (rG4) structural information datasets from the G4Atlas database (38), RBP binding datasets from the POSTAR3 database (39), RNA loop information datasets from the R-loopAtlas database (40), transcriptome-scale RNA secondary structure probing datasets from the RNA Atlas of Structure Probing (RASP) database (41), APA site datasets from the PlantAPAdb database (42), small open reading frame (sORF) datasets from the PsORF database (http://psorf.whu.edu.cn), conservation datasets from the PlantRegMap database (43) and known RNA modification-related enzyme datasets from the Modomics database (44).

### Plant material, RNA extraction and sequencing for wild rice and cultivated rice

The MeRIP-seq analysis was performed for *Oryza rufipogon* (DXW81; common wild rice) and the following two cultivated rice subspecies: *Oryza sativa* ssp. *indica* (WSSM) and *Oryza sativa* ssp. *japonica* (ZH11).

- Plant material and growth conditions: DXW81, WSSM and ZH11 were grown in the experimental field of the Rice Research Institute, Guangdong Academy of Agricultural Sciences, Guangzhou, China. Young leaves at the tillering stage and panicles at the booting stage were collected for the MeRIP-seq analysis. All tissues were immediately frozen in liquid nitrogen and stored at −80°C prior to the RNA extraction.
- RNA extraction and sequencing: Total RNA was isolated and purified using the TRIzol reagent (Invitrogen, CA, USA) following the manufacturer's procedure. The RNA quantity and purity were determined using the NanoDrop ND-1000 spectrophotometer (NanoDrop, USA), whereas RNA integrity (RNA integrity number > 7.0) was assessed using the Bioanalyzer 2100 system (Agilent, CA, USA) and confirmed by denaturing agarose gel electrophoresis. An A-base was added to the blunt ends of each strand to facilitate the ligation to the index adapters, which had a T-base overhang. Single- or dual-index adapters were ligated to the fragments, after which a size selection step was completed using AMPureXP beads. After the heat-labile UDG enzyme (cat.m0280; NEB, USA) treatment of the U-labeled double-stranded DNA fragments, the ligated products were amplified by PCR under the following conditions: initial denaturation at 95°C for 3 min; eight cycles of denaturation at 98°C for 15 s, annealing at 60°C for 15 s and extension at 72°C for 30 s; and then final extension at 72°C for 5 min. The average insert size for the final cDNA library was 300 ± 50 bp. Finally, we performed a paired-end sequencing analysis (2 × 150 bp) using the Illumina NovaSeq™ 6000 system (LC-Bio Technology Co., Ltd, Hangzhou, China).

### Data preprocessing

The MeRIP-seq datasets in the SRA format were converted to the FASTQ format using sratoolkit v2.11.0 (https://github.com/ncbi/sra-tools) and then the default parameters of fastp v0.20.0 (45) were used to trim the adapter sequences and low-quality bases. After filtering the data for quality, STAR 2.7.4a (46) was used to map clean reads to the corresponding reference genomes. The parameter settings were as follows: –outSAMtype BAM SortedByCoordinate –outFilterMultimapNmax 1 –outFilterMismatchNmax 2 –runThreadN 16 –readFilesCommand zcat –outFileNamePrefix.

### Peak calling and m^6^A level calculation

Using the BAM format genome mapping files of the IP and the input samples obtained as described above, we applied two peak calling strategies. The following parameters of MACS2 (47) were used to identify methylation peaks: –nomodel –extsize 150 -B -n -q 0.05. The R package exomePeak2 (48) (https://github.com/ZW-xjtlu/exomePeak2) was used for peak calling, which involved the same BAM files and GTF files. The parameters were set as follows: fragment_length = 100 binding_length = 25 step_length = 25 pc_count_cutoff = 5 bg_count_cutoff = 50 p_cutoff = 1e-5 peak_calling_mode = exon. The m^6^A level for each peak was calculated on the basis of RNASeQC v2.4.2 (49), bedtools v2.17.0 (50) and our in-house pipelines as:


\begin{equation*}{{\mathrm{m}}}^6{\mathrm{A}}\,level = \frac{{{\mathrm{Peak}}\,{\mathrm{reads}}\,({\mathrm{IP}}) \times {\mathrm{Total}}\,{\mathrm{reads}}\,({\mathrm{Input}})}}{{{\mathrm{Peak}}\,{\mathrm{reads}}\,({\mathrm{Input}}) \times {\mathrm{Total}}\,{\mathrm{reads}}\,({\mathrm{IP}})}}\end{equation*}

### Prediction of m^6^A sites

The cDNA and ncRNA sequences of the following plant species were downloaded from EnsemblPlants: Aegilops tauschii, A. thaliana, Brassica rapa, Glycine max, Malus domestica, O. sativa, Physcomitrium patens, Populus trichocarpa, Phaseolus vulgaris, Sorghum bicolor, Solanum lycopersicum, Triticum aestivum, Triticum dicoccoides and Zea mays. The cDNA and ncRNA sequences of the following five species were obtained from the databases mentioned above: *F. vesca, G. arboreum, G. hirsutum, N. benthamiana* and *P. fortunei*. The SRAMP (16) software was used to predict all potential m^6^A sites.

### Identification of orthologous genes among plant species and analysis of evolution

To analyze orthologous groups (OGs), the longest protein was identified by examining the protein-coding sequences. The OrthoFinder v2.4.0 software (51) was used to construct OGs among 20 plant species on the basis of the longest protein. We also investigated the evolutionary patterns of m^6^A methylation divergence. The ParaAT v2.0 software (52) was used for the sequence alignment analysis involving the longest protein sequences, after which KaKs_calculator v2.0 (53) (with the Model Averaging method) was used to calculate the nonsynonymous substitutions-to-synonymous substitutions (Ka/Ks) ratio.

### Analyses of alternative polyadenylation, expression levels, differential usage of exons and translational efficiency

The BAM files for the MeRIP-seq inputs (RNA-seq) were used to analyze gene expression, APA and exon usage. The featureCounts program in the Subread package (54) was used to calculate gene read counts, which were then normalized to RPKM (reads per kilobase per million mapped reads) values. DaPars2 was used to infer the dynamic APA for each sample (55,56). The DEXSeq (57) package was used to examine differential exon usage on the basis of RNA-seq exon counts between samples examined using different experimental methods. The TE of each gene was calculated as ‘RPKM (translation level)/RPKM (expression level)’ on the basis of the ribosome profiling (Ribo-seq) datasets of four species (*A. thaliana*, *O. sativa*, *S. lycopersicum* and *Z. mays*), which were obtained from the NCBI SRA database.

### Construction and analysis of co-methylated m^6^A gene network

We evaluated the variant m^6^A peaks to construct the m^6^A gene co-methylation network using the WGCNA package (58) implemented in R. We constructed an adjacency matrix to describe the extent of the correlation of the m^6^A levels between genes using the ‘adjacency’ function (scale-free R2 > 0.9). Subsequently, a topological overlap matrix (TOM) was derived from the adjacency matrix using the ‘TOMsimilarity’ function:


\begin{equation*}\mathrm{TO}{\mathrm{M}}_{ij} = \frac{{\sum_{u \ne i,j}({a}_{iu}*{a}_{uj}) + {a}_{ij}}} {\min \lbrace{\mathrm{k}}_i,\ {\mathrm{k}}_{j}\rbrace + 1 - {a}_{ij}}\end{equation*}

Finally, we calculated the eigengene, hierarchically clustered the modules and merged similar modules.

### Trait ontology enrichment analysis of the m^6^A gene co-methylation module

We conducted a trait ontology (TO) enrichment analysis to verify the gene modules that were enriched with agronomic trait-associated genes after obtaining the gene co-methylation modules. The p-value was calculated according to the hypergeometric distribution as previously described (59):


\begin{equation*}p{\mathrm{\ }}{\left( {TO,{\mathrm{\ }}module} \right)}^{\mathrm{\ }} = \mathop \sum \limits_{x = m}^{\min \left( {n,k} \right)} {\mathrm{\ }}\frac{{\left( {\begin{array}{@{}*{1}{c}@{}} {\mathrm{k}}\\ {\mathrm{x}} \end{array}} \right)\left( {\frac{{N - K}}{{n - x}}} \right)}}{{\left( {\begin{array}{@{}*{1}{c}@{}} N\\ n \end{array}} \right)}}\end{equation*}

where *N* represents the total number of genes, *K* represents the total number of genes assigned a TO term among all genes, *n* represents the number of genes in the module and *m* represents the number of genes assigned a TO term in the module. The modules with adjusted p-values less than 1e-3 were considered to be gene modules associated with the corresponding agronomic trait.

### Nucleotide-binding leucine-rich repeat gene family identification and classification

We used previously described methods (60) to identify and classify nucleotide-binding leucine-rich repeat (NLR) gene families. Briefly, InterProScan (61) (with E-value < 1e-4) was used to identify the genes containing the NB-ARC domain as candidate genes. Next, NLR-Parser (version 3.0) (62) was used to determine the LRR domain, and the results were cross-verified with InterProScan. Finally, the candidate genes with any of motif 9, motif 11, or motif 19 in NLR-Parser were designated as NLR genes in rice. The NLR gene family members were divided into the following three categories according to their distribution on chromosomes: singleton genes, gene pairs and gene clusters.

### Identification of genes encoding plant RNA modification-related enzymes

All genes encoding known RNA modification-related enzymes were obtained from the Modomics database. The encoded enzyme sequences in 20 plant species were extracted from the respective protein reference sequences. The Blastp program of blast-2.2.26 (63) was used to align sequences and then the best bidirectional hits were used to obtain the orthologous enzyme-encoding genes for the 20 plant species. In addition, the expression profiles of the identified RNA modification-related enzyme-encoding genes in different tissues were obtained from the Plant Public RNA-seq Database (http://ipf.sustech.edu.cn/pub/plantrna/) and displayed using the Plotly package (https://github.com/plotly/plotly.R). Moreover, protein–protein interactions among the enzymes encoded by these genes were determined using the STRING database (https://cn.string-db.org/) and displayed using RNAmodNet developed in this study. Because of the lack of related data, the current version of PRMD provides the RNA modification-related gene expression patterns for only six species and the protein–protein interactions for only three species.

### Annotation of RNA modifications

RNAmod (24), which is an interactive and freely available platform for the annotation and visualization of RNA modifications, was used to annotate the RNA modifications in PRMD. First, RNAmod extracted gene features, such as promoter regions, 5′ and 3′ untranslated regions (UTRs), start codon regions, coding sequence (CDS) regions and stop codon regions, from different annotated reference genomes and then examined gene characteristics, including the GC content, length and minimum free energy. Second, RNAmod mapped all modification sites to different RNA features and calculated coverage values and analyzed metagenes and other annotations. The modified genes were functionally characterized on the basis of Gene Ontology (GO) (64) and KEGG pathways (65) using the clusterProfiler package (66) and according to Reactome pathways (67) using ReactomePA packages (68).

### Database and web interface implementation

MySQL was used for storing and managing metadata in PRMD. The PHP/Apache environment in a Linux system equipped with 512 GB RAM and four Octa-core AMD processors (2.6 GHz each) was used to host the PRMD web server. The back-end workflow and plots were implemented using the Python/Perl language and R packages. Additionally, JQuery, DataTable, Highchart and igv.js were embedded in the system for dynamic and interactive data visualizations (69,70). A web-based genome browser was implemented using JBrowse (71). mRNAbrowse was designed for visualizing RNA modifications on the basis of igv.js (https://github.com/igvteam/igv.js), whereas RNAlollipop was designed to develop lollipop views of RNA modifications.

## Database content and usage

### Overview of the PRMD content

We developed PRMD as a comprehensive resource for information regarding RNA modifications. For m^6^A, 693 MeRIP-seq samples were carefully collected from the SRA and GSA databases for the following 19 plant species: *Ae. tauschii* (6), *A. thaliana* (259), *B. rapa* (24), *F. vesca* (18), *G. max* (24), *G. arboreum* (6), *G. hirsutum* (18), *M. domestica* (32), *N. benthamiana* (18), *O. sativa* (88), *P. fortunei* (8), *P. patens* (6), *P. trichocarpa* (26), *P. vulgaris* (6), *S. bicolor* (22), *S. lycopersicum* (38), *T. aestivum* (22), *T. dicoccoides* (6) and *Z. mays* (66). In addition, 12 MeRIP-seq samples were generated in this study for *O. rufipogon* (DXW81), *O. sativa* ssp. *indica* (WSSM) and *O. sativa* ssp. *japonica* (ZH11). The overall statistical analysis of all datasets indicated the m^6^A methylation ratios were negatively correlated with the genome size and the number of genes in these plant species (Supplementary Figure S1).

**Figure 1.**
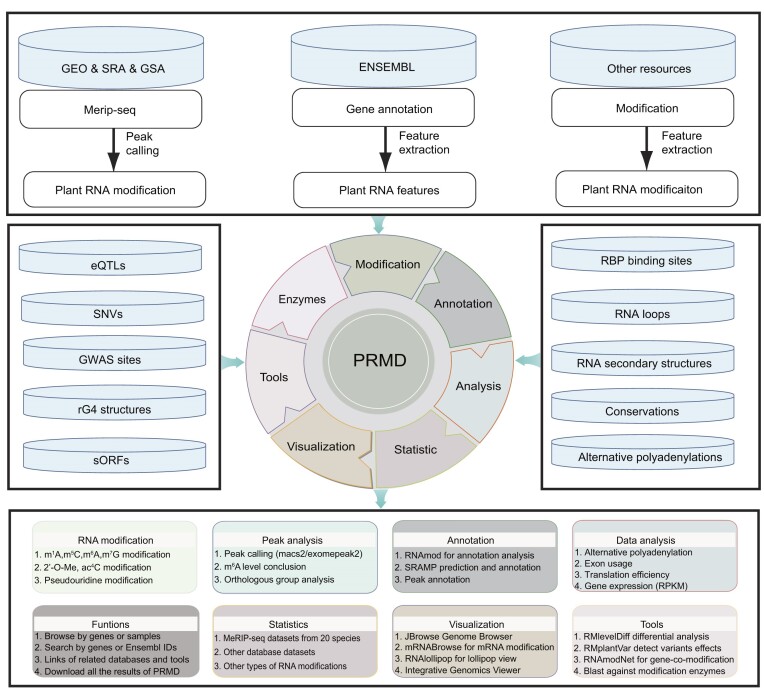
Overall workflow of PRMD. PRMD provides comprehensive information about RNA modifications. All of the data generated by PRMD were deposited in a MySQL database and displayed in several convenient modules on web pages. In addition to the m^6^A modification, PRMD also contains other types of known RNA modifications (m^1^A, m^5^C, m^7^G, ac^4^C, 2′-O-Me and pseudouridine) and additional related datasets derived from published research articles and related plant resources. Furthermore, PRMD provides several convenient tools for visualizing and analyzing data.

Moreover, PRMD integrated datasets of other types of RNA modifications, such as m^1^A, m^5^C, m^7^G, ac^4^C, 2′-O-Me and pseudouridine, as well as additional related datasets, including those for eQTLs, SNVs, GWAS sites, rG4 structures, sORFs, RBP binding sites, RNA loops, RNA secondary structures, conservations and APA site information. All datasets were processed through our uniform pipelines. The information was deposited in a MySQL database and displayed in convenient web modules in PRMD. Furthermore, we designed PRMD to enable users to easily visualize and analyze the data in the database (Figure 1).

### Web interface modules developed in PRMD

**Figure 2.**
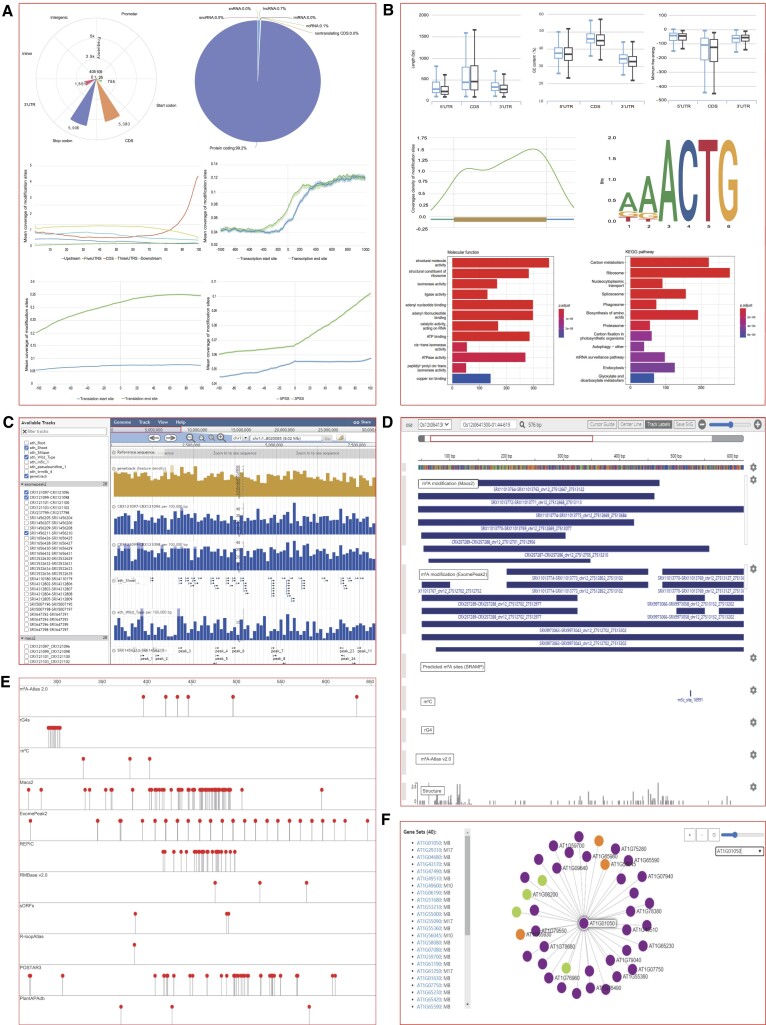
Main functions of the PRMD database. (**A**) Outputs of statistical analyses, including peak gene feature distribution, gene type distribution, coverage plots for different features, boundary coverage for transcription start/end sites, boundary coverage for translation start/end sites and boundary coverage for splice sites. (**B**) Outputs of annotation analyses, including RNA characteristics of genes with modifications, mRNA metagene plot, enriched motifs, enriched Gene Ontology terms among the modified genes and enriched functional pathways among the modified genes. (**C**) Visualization of JBrowse data. JBrowse displays tracks of RNA modifications and other related annotations with genomic coordinates. (**D**) Visualization of mRNAbrowse data. mRNAbrowse shows tracks of RNA modifications and other related annotations. (**E**) Visualization of RNAlollipop data. RNAlollipop was designed to produce lollipop views of RNA modifications. (**F**) Outputs of the gene co-methylation network analysis. Dots represent gene names, whereas lines represent protein–protein interactions. Because of the limited number of samples, only four species are currently available for gene co-methylation analyses.

- The ‘Browse’ module contains the following five tables: (i) browse by genes; (ii) browse by samples; (iii) other modifications; (iv) predicted m^6^A sites and (v) RNA modification-related enzymes. The ‘Browse by genes’ table contains information about the genes with m^6^A modifications (with hyperlinks for gene details), list of supported samples, list of m^6^A orthologous genes and other visualization tools (e.g. mRNAbrowse and JBrowse). The ‘Browse by samples’ table consists of information about the MeRIP-seq samples (with hyperlinks for sample details in the original database), references and the RNA modification annotation module. The ‘Other modifications’ table contains other types of RNA modifications (Supplementary Table S4), with hyperlinks for the original resource and modification annotation module. The ‘Predicted m^6^A sites’ table displays the predicted m^6^A sites for 20 species (Supplementary Table S5), with hyperlinks for the annotation module for each species. The ‘RNA modification-related enzymes’ table includes a list of the genes encoding the RNA modification-related enzymes identified in 20 plant species (with hyperlinks for gene details), protein–protein interaction network comprising RNA modification-related enzymes and expression profiles of the genes encoding RNA modification-related enzymes in different tissues.
- The ‘Search’ module in PRMD allows users to quickly obtain comprehensive information by submitting gene IDs, transcript IDs, gene symbols, sample IDs, study IDs, or PubMed IDs. We also added a quick-search function that supports one query on the PRMD homepage.
- The ‘Annotation’ module provides details regarding RNA modifications, including the following: (a) overall statistics for the modifications across different RNA features and RNA biotypes; (b) density/histogram map of RNA modifications at RNA exon splice junctions, RNA transcription start/end sites and RNA translation start and stop codons; (c) identified motifs at RNA modification sites; (d) RNA modification site heatmap; (e) enriched GO terms and KEGG pathways among the modified genes and (f) metagene analysis in a specific gene context (Figure 2A, B). This module can be used to present RNA modification features in each sample. For example, m^6^A sites were mainly located in the CDS and 3′ UTRs, with a density peak near the translation end sites. In contrast, the other types of modifications, including m^5^C and pseudouridine, were similarly distributed, with density peaks near the translation start sites (Supplementary Figure S2).
- The ‘Visualization’ module contains the following four visualization tools: (a) JBrowse embedded in a fast, scalable genome browser built using JavaScript and HTML5 to visualize all RNA modification sites and other publicly available annotations (genome scale) (Figure 2C); (b) mRNAbrowse, which intuitively displays RNA modifications (transcript scale) (Figure 2D); (c) RNAlollipop, which displays the lollipop view of merged datasets containing the transcript locations (Figure 2E) and (d) IGV, which visualizes the read distribution of modification peaks on the basis of the BigWig format files (genome scale).
- The ‘Tools’ module contains the following four convenient analytical tools: (a) RMlevelDiff for analyzing RNA modification levels and differential modifications; (b) RMplantVar for detecting potential deleterious variant effects on RNA modifications; (c) RNAmodNet for analyzing gene co-methylation networks and visualizing m^6^A co-modification gene networks (Figure 2F) and (d) Blast for identifying potential RNA modification-related enzymes among the sequences uploaded by users.
- The other modules include the following: (a) the ‘Statistics’ module, which contains the overall statistics for RNA modifications among species; (b) the ‘Download’ module, which provides the datasets of different types of RNA modifications and different species, with users able to download data with one click depending on the species and type of RNA modification; (c) the ‘Links’ module, which provides dozens of software and database links for RNA modifications and (d) the ‘Document’ module, which provides a detailed explanation for the data processing.

### Visualization tools developed in PRMD

mRNAbrowse was designed for the intuitive visualization of RNA modifications and related datasets (transcript scale), including modification sites determined by nanopore sequencing and miCLIP-seq, sequence conservation, GWAS sites, miRNA target sites, APA sites, RBP binding sites, RNA secondary structures, rG4 structures, R-loop elements, sORFs and other types of modifications. In mRNAbrowse, users can zoom in and out using buttons in the upper right corner to visualize the modification site sequence context.

RNAlollipop was designed to establish lollipop views of the modifications and other datasets in PRMD. For each dataset type, all of the datasets were merged to enable users to intuitively compare the RNA modifications with other elements at the same location from different sources. JBrowse was integrated to visualize the modification sites and other information (genome scale). In addition, IGV, with hyperlinks provided in the table with specific details and information, was integrated to visualize the distribution of the modification peaks on the basis of the BigWig files of the input and IP samples in the genome. Users can intuitively check the read coverage of the modification peaks called by MACS2 and exomePeak2 among different samples.

### Web-based analytical tools developed in PRMD

**Figure 3.**
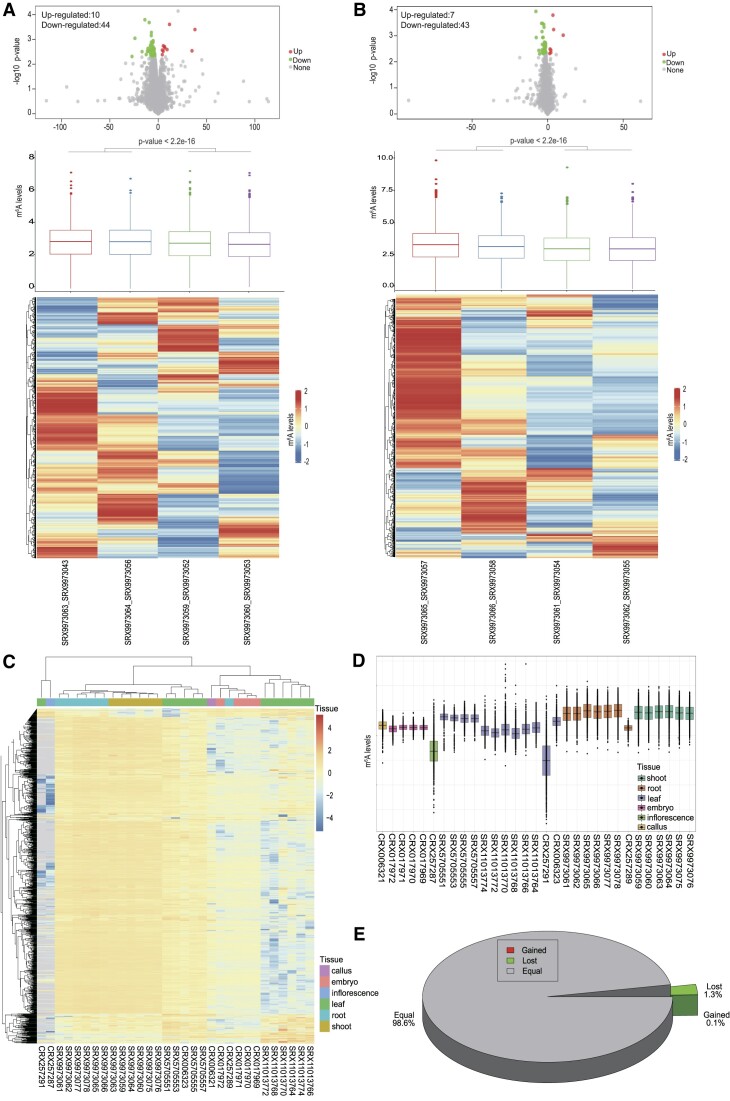
Comparative analysis of m^6^A modifications in *Oryza sativa*. (**A**, **B**) RMlevelDiff comparison of m^6^A levels between samples. (**C**) Pheatmap of m^6^A levels. (**D**) Boxplot of m^6^A levels. (**E**) RMplantVar outputs, including a pie chart and a table.

RMlevelDiff was designed to analyze the differences in m^6^A levels; the parameter settings are provided in Supplementary Figure S3. When users select a species, all of the MeRIP-seq samples for that species will be displayed. Users must divide their samples of interest into different groups to perform the analysis. Notably, each group should contain at least one sample. The outputs include the following: (a) a volcano plot of the differences in modification levels, with red and green points representing up-regulated and down-regulated modification sites, respectively; (b) a box plot and a heatmap plot showing the distribution of m^6^A levels among samples; (c) a detailed list of m^6^A modification peaks in selected samples and the log_2_(fold-change) value, *P*-value and false discovery rate and (d) a detailed list of the means and fold-changes of the two groups of selected samples. Using *O. sativa* (Accession: SAMN19341036) as an example, the shoot and root tissue samples were divided into two groups, with each group comprising two pairs of FTO transgenic and wild-type samples. The RMlevelDiff analysis indicated that the shoot (FTO transgenic versus wild-type) had 10 up-regulated and 44 down-regulated peaks, whereas the root (FTO transgenic versus wild-type) had 7 up-regulated and 43 down-regulated peaks. For the shoot and the root, the FTO transgenic samples had a significantly higher m^6^A methylation ratio (*P*-value < 2.2e − 16) than the wild-type samples (Figure 3A–D).

RMplantVar was designed to detect potential variants affecting RNA modifications. A variant file in the VCF format and the objective sample list from PRMD are used as the inputs for RMplantVar (Supplementary Figure S3). After users submit the data, the queue system will provide a job ID, which can be used to check the progress of the analysis and retrieve the results. Moreover, users have the option of being notified by email when their job has been completed. The results include a pie chart presenting the distribution of the m^6^A lost, m^6^A gained and unchanged variants as well as a table with specific details, including the location of variants, transcript IDs, peak IDs, reference motifs, mutated motifs and the score used to determine whether the variants were lost or gained. Using the variations from the Rice SNP-Seek Database (https://snp-seek.irri.org/_download.zul) as an example, RMplantVar analyzed the sequence characteristics of the sites with mutated RNA modifications. Approximately 1.3% of the mutation sites were designated as m^6^A lost variants, whereas 0.1% of the mutation sites were designated as m^6^A gained variants. Accordingly, these mutation sites altered the DRACH motif structure, which may influence the binding of writers/readers/erasers (Figure 3E and Supplementary Table S6).

RNAmodNet was developed to visualize the m^6^A co-modification gene network. PRMD currently supports four species with many samples (>30), including *A. thaliana*, *O. sativa*, *S. lycopersicum* and *Z. mays*. Users are required to select a species and a gene from the input parameters. All genes associated with the selected gene will be displayed, with different colors used to represent different network modules. Additionally, gene names and their modules will be displayed on the left panel. Specific gene details can be obtained by clicking the gene name.

Basic Local Alignment Search Tool (Blast) was integrated in PRMD to screen for genes encoding known RNA modification-related enzymes. Users can upload protein sequences in the FASTA format and input other related Blast parameters. The results consist of a table that lists query sequence IDs, gene IDs of known RNA modification-related enzymes, percentage of identity, alignment length, e-values and the start/end of the query and subject. These genes encoding RNA modification-related enzymes can be used to search for particular RNA modifications and perform downstream functional studies.

## Case studies

**Figure 4.**
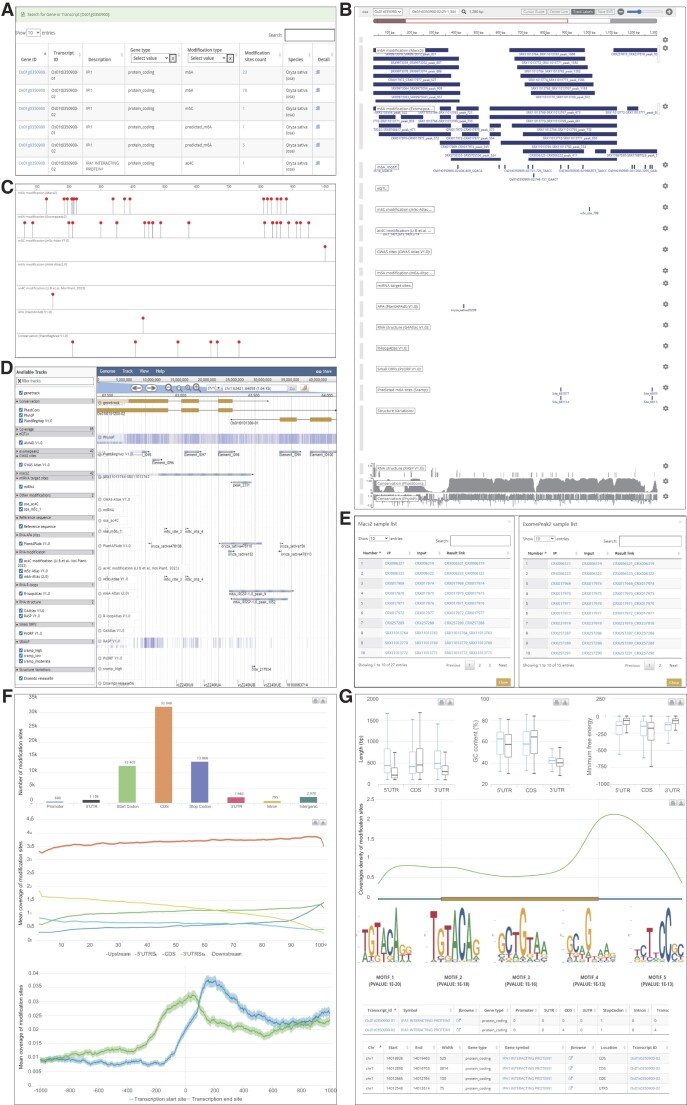
Comprehensive analysis of *IPA1* in PRMD. (**A**) Outputs of the ‘Search’ module in PRMD revealed that *IPA1* produces two transcripts with differing RNA modifications (m^6^A and m^5^C). (**B**) mRNAbrowse displays the tracks of mRNA modification peaks derived from different methods and different sources. (**C**) Lollipop view of RNA modifications. (**D**) JBrowse shows the tracks of m^6^A RNA modifications and other related annotations. (**E**) Details regarding *IPA1*. (**F**) Various annotations, including peak gene feature and type distributions, coverage plots for different features and boundary coverage for transcription start/end sites. (**G**) RNA characteristics of genes with modifications, RNA metagene plots, enriched motifs, enriched pathways and gene annotation information are presented in a tabular form.

In rice, *IDEAL PLANT ARCHITECTURE INTERACTING PROTEIN 1* (*IPI1*) is an important gene that encodes a protein that modulates IPA1 protein levels, thereby regulating the plant architecture (72). The two transcripts derived from this gene differ in terms of two RNA modifications (m^6^A and m^5^C) in PRMD. The ‘Browse’ module revealed that the m^6^A of the transcript Os01t0350900-01 is supported by 27 MACS2 and 15 exomePeak2 samples, whereas the m^6^A in the other transcript (Os01t0350900-02) is supported by 37 MACS2 and 32 exomePeak2 samples. Additionally, mRNAbrowse displayed the tracks of the RNA modification peaks and different related sites (transcript scale), including m^5^C modification sites, predicted m^6^A sites and an APA site in the PlantAPAdb database. RNAlollipop presented the distribution of the modification sites and other related information for whole transcripts, whereas JBrowse showed the tracks of the m^6^A RNA modifications and other related sites (genome scale), including m^5^C modification sites and predicted m^6^A sites. Furthermore, 43 orthologous m^6^A-modified genes were detected among 20 plant species (Figure 4A–E).

The m^6^A modification sites were highly enriched in the CDS. The GC content was higher in the CDS than in the UTRs, whereas the opposite trend was detected for the minimum free energy. The metagene analysis of RNA modifications indicated that m^6^A was enriched near the stop codon and the consensus motif ‘DRACH’ was enriched (Figure 4F, G). We further analyzed the distribution of the m^6^A peaks in IGV using the genome mapping files, which revealed that m^6^A modifications occurred at the same location in the genomes of different samples. The *IPI1* homolog in *A. thaliana* (AT3G05545) had a similar distribution in IGV (Supplementary Figure S4), indicative of conserved m^6^A modifications between rice and *A. thaliana*.

## Comparative analyses using the data in PRMD

### Orthogroup gene identification, evolution analyses

In total, 47671 orthogroups were identified in 20 plant species. We subsequently selected four species with more than 30 samples (*A. thaliana*, *O. sativa*, *S. lycopersicum* and *Z. mays*) to perform a comparative analysis, which detected 1126 (*A. thaliana*), 1252 (rice), 1218 (tomato) and 1608 (maize) species-specific orthogroups and 9715 common orthogroups. The comparison between *A. thaliana* and rice identified 2881 one-to-one orthogroups with m^6^A modifications. Synonymous and nonsynonymous substitutions among these orthogroup genes revealed that the evolutionary rate was significantly lower for both m^6^A-modified genes than for the non-m^6^A-modified genes (*P*-value < 2.2e−16), indicating that the genes with m^6^A modifications were under purifying selection during evolution (Supplementary Figure S5A–C).

### Gene co-methylation network analyses of rice and *arabidopsis thaliana*

**Figure 5.**
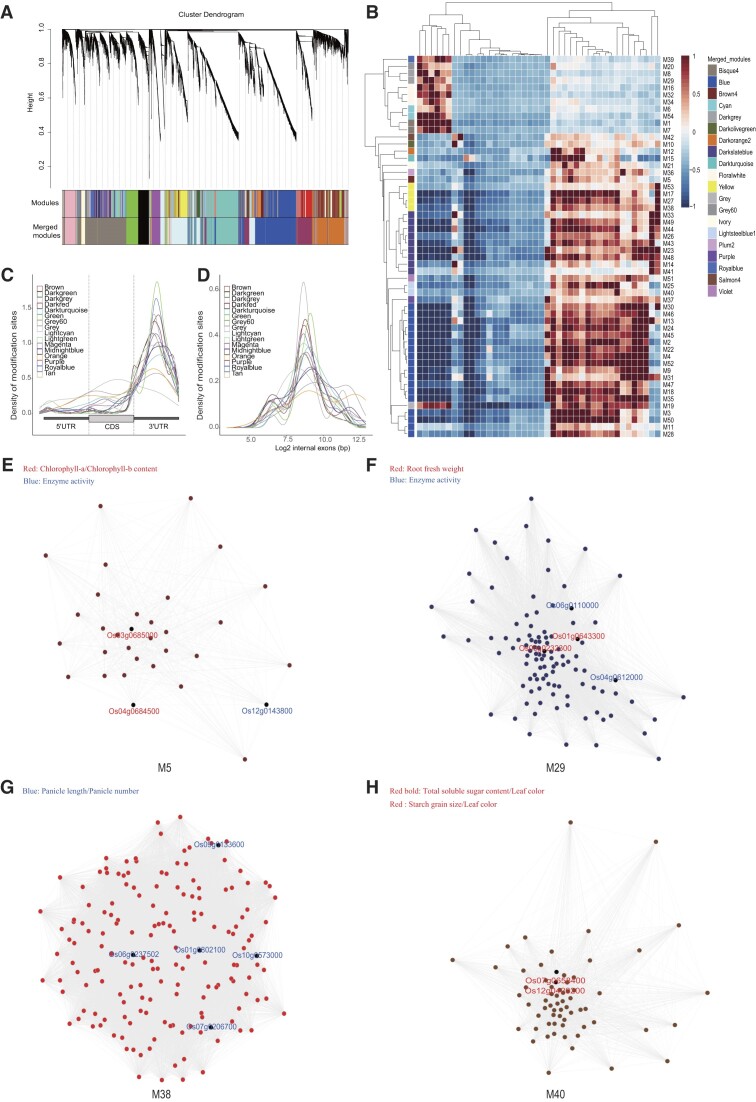
Classification and analyses of the co-methylation m^6^A modules in *Oryza sativa*. (**A**) Classification of co-methylated m^6^A modules. (**B**) Heatmap presenting the m^6^A indices of all co-methylation modules across all rice samples. (**C**) Density distributions of m^6^A peaks in different combined modules across the 5′ UTR, CDS and 3′ UTR. (**D**) Density distributions of log-transformed lengths of the internal exons with m^6^A peaks in different combined co-methylation modules. (**E–H**) In *Oryza sativa*, the following modules were associated with several traits: M5 (chlorophyll content and enzyme activity), M29 (root fresh weight and enzyme activity), M38 (panicle length/panicle number) and M40 (total soluble sugar content, leaf color and starch grain size).

We obtained 20 and 16 modules in the m^6^A co-methylation networks of *O. sativa* and *A. thaliana*, respectively, on the basis of the correlations between m^6^A indices (Figure 5A, B and Supplementary Figure S6A, B). The metagene analysis of the m^6^A peak distribution in different modules indicated that m^6^A peaks were strongly enriched in the 3′ UTR in rice, which was consistent with the results of an earlier study by Yu *et al.* (73). The lengths of the internal exons with m^6^A were similarly distributed in the co-methylation modules of *O. sativa* (Figure 5C, D). In contrast, in *A. thaliana*, m^6^A was enriched in the CDS and 3′ UTR and the internal exon length distribution varied among the co-methylation modules (Supplementary Figure S6C, D), suggesting the m^6^A methylomes had species-specific dynamic topologies.

We also explored the distribution of m^6^A-modified genes that were associated with different traits in *O. sativa*. More than 50% of the m^6^A-modified genes were related to responses to biotic and abiotic stresses as well as quality and yield (Supplementary Figure S5D), reflecting the influence of RNA modifications on these important traits. We performed TO analyses to explore the m^6^A co-methylation modules significantly associated with these important traits. In rice, several modules closely related to specific traits were identified, including M5 (chlorophyll content and enzyme activity), M29 (root fresh weight and enzyme activity), M38 (panicle length/panicle number) and M40 (total soluble sugar content, leaf color and starch grain size) (Figure 5E–H). Notably, M40 was a broadly expressed module associated with three different traits, indicative of the functional diversity of the genes in this module. However, in *A. thaliana*, modules M2, M10, M48 and M55 were related to panicle size, male sterility/starch content, sucrose content/sugar content and male sterility, respectively (Supplementary Figure S6E–H), indicative of the conservation of the m^6^A co-methylation modules for these traits. Interestingly, M38 of *O. sativa* and M2 of *A. thaliana* were associated with the panicle, implying different gene modules may have similar functions in different species.

### Comparative analysis of *oryza rufipogon* and two cultivated rice subspecies (*oryza sativa* ssp. *Indica* and *oryza sativa* ssp. *Japonica*)

**Figure 6.**
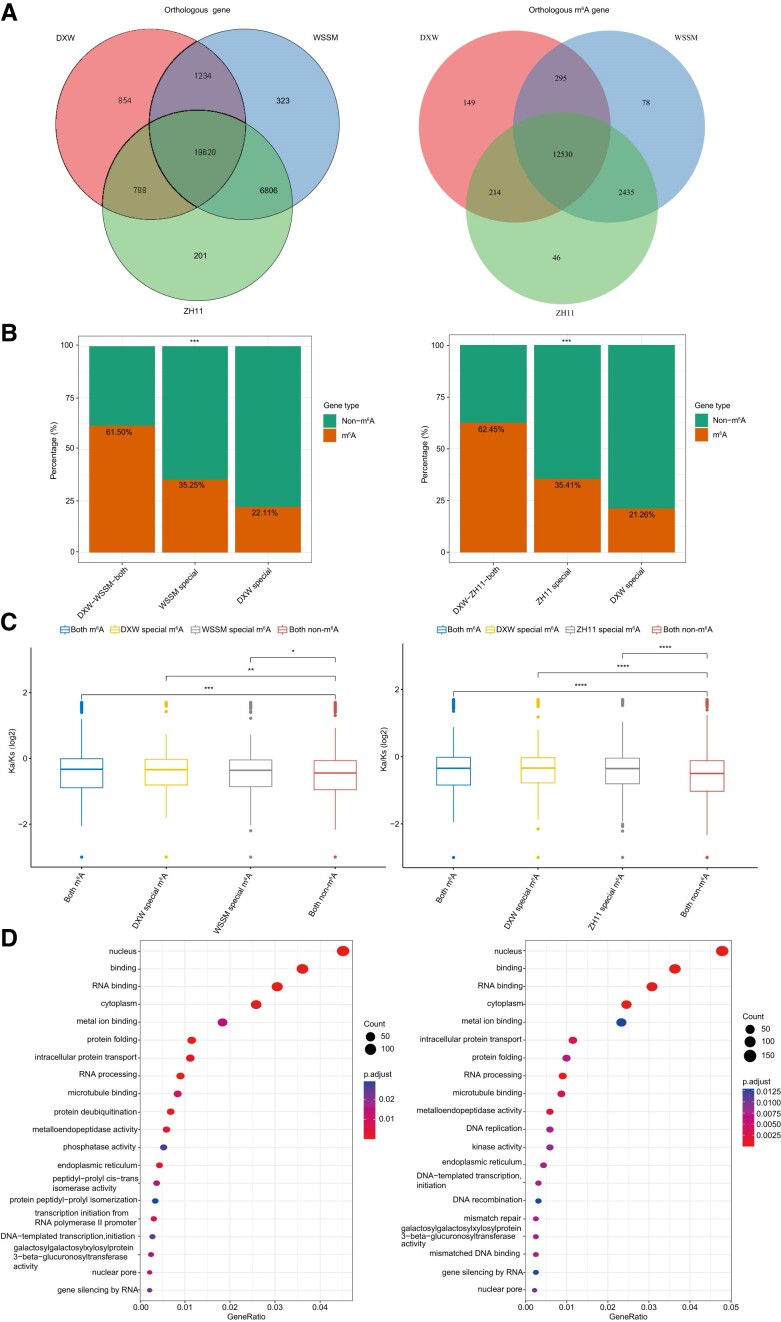
Analysis of wild rice (*Oryza rufipogon*) and two cultivated rice subspecies (*Oryza sativa* ssp. *indica* and *Oryza sativa* ssp. *japonica*). (**A**) Number of orthologous genes and m^6^A-modified genes among *O. rufipogon*, *O. sativa* ssp. *indica* and *O. sativa* ssp. *japonica*. (**B**) m^6^A methylation ratios for the one-to-one orthologous gene pairs of DXW81 versus WSSM and DXW81 *vs* ZH11. (**C**) Ka/Ks values for the one-to-one orthologous gene pairs of DXW81 versus WSSM and DXW81 *vs* ZH11. (**D**) GO enrichment analysis of the one-to-one orthologous gene pairs of DXW81 versus WSSM and DXW81 versus ZH11.

The MeRIP-seq datasets derived from our sequencing analyses of *O. rufipogon* (DXW81), *O. sativa* ssp. *indica* (ZH11) and *O. sativa* ssp. *japonica* (WSSM) were selected for the comparative analysis. The m^6^A peak density distributions were consistent with the earlier observations that m^6^A accumulated substantially around the stop codon and within the 3′ UTR and that the m^6^A methylation ratio among all genes was higher for both WSSM and ZH11 than for DXW81 (Supplementary Figure S7A, B). Orthologous group analyses detected 19620 orthogroups that had a high m^6^A methylation ratio in DXW81, WSSM and ZH11. The DXW81 *vs* WSSM and DXW81 versus ZH11 comparisons indicated that the m^6^A methylation ratio was significantly higher for the one-to-one orthologous gene pairs (DXW81–WSSM and DXW81–ZH11) than for the species-specific m^6^A-modified genes (*P*-value < 2.2e−16; Fisher's exact test). Synonymous and nonsynonymous substitutions among these orthogroup genes suggested that m^6^A-modified genes were likely under purifying selection during evolution (Figure 6A–C). The common enriched GO terms (DXW81 and WSSM as well as DXW81 and ZH11) among the orthologous genes with m^6^A modifications included metalloendopeptidase activity, DNA-templated transcription initiation and galactosylgalactosylxylosylprotein 3-beta-glucuronosyltransferase activity, suggestive of the relative similarity in these m^6^A-modified genes between the two cultivated rice subspecies (Figure 6D).

We also identified NLR gene families in DXW81, WSSM and ZH11, and then divided them into three categories (singleton genes, gene pairs and gene clusters). The m^6^A methylation ratios were higher for the gene pairs and gene clusters than for the singleton genes. Moreover, the m^6^A methylation ratios for the gene pairs and gene clusters were higher for WSSM and ZH11 than for DXW81 (Supplementary Figure S7C). These results imply m^6^A methylations may have been important for rice domestication.

## Comparison with other databases

Several databases and web servers that integrate the existing sequencing data have been constructed to help researchers identify diverse RNA modifications. However, most of these databases were focused on mammalian species, especially human and mouse (Table 1). For example, m6AVar (74) and RMVar (75) focus on m^6^A-associated variants that potentially affect RNA modifications in human and mouse. The MeT-DB v2.0, REPIC and RMBase v2.0 databases contain only one type of RNA modification (m^6^A) for one plant species (*A. thaliana*). The m6A-Atlas database (76) comprises 442162 m^6^A sites identified from the epitranscriptome profiles of several species (e.g. human, mouse, fly, zebrafish, rat, yeast, *A. thaliana* and virus); the updated version of m6A-Atlas (i.e. v2.0) (23) includes only six plant species (*A. thaliana*, black cottonwood, maize, tomato, rice and wild strawberry), with 172 MeRIP-seq samples for *A. thaliana* (78), *F. vesca* (18), *O. sativa* (8), *S. lycopersicum* (18) and *Z. mays* (24). The ENCORE database is an updated version of RMBase that includes 10 plant species, but m^6^A modifications are provided only for *A. thaliana* and there are relatively few samples for different modifications. In addition, there are single-species databases, such as AthMethPre (18) and RFAthM6A (19), which are only used for predicting m^6^A sites in *A. thaliana*. Most importantly, the available databases lack comprehensive annotations and cannot intuitively visualize plant RNA modifications and other related data.

**Table 1.** Comparison with other integrated RNA modification related databases

Name	PRMD	m^6^A-Atlas 2.0	MeT-DB 2.0	REPIC	RMBase v2.0	ENCORE
Data resources of plants						
Species	20	6	1	1	1	10
Modification types	m^1^A, m^5^C, m^6^A, m^7^G, ac^4^C, 2′O-Me, Pseudo	m^6^A	m^6^A	m^6^A	m^6^A	m^1^A, m^5^C, m^6^A, m^7^G, 2′O-Me, Pseudo
m^6^A-seq	693	156	42	36	16	16
Other types modification sites	79973	No	No	No	No	2762
Nanopore-seq	Yes	No	No	No	No	No
miCLIP-seq	Yes	Yes	Yes	No	No	Yes
Other plant specific regulation datasets						
RBP binding sites	Yes	Yes	No	No	No	Yes
RNA Structures	Yes	No	No	No	No	No
micro-RNA target sites	Yes	No	No	No	No	No
Single nucleotide polymorphisms (SNPs)	Yes	Yes	No	No	No	No
Expression Quantitative Trait Loci (eQTLs)	Yes	No	No	No	No	No
GWAS sites	Yes	No	No	No	No	No
Small open reading frames (sORFs)	Yes	No	No	No	No	No
Tools						
Differentially methylated analysis	Yes	Yes	No	No	No	No
Variation effects on modifications	Yes	No	No	No	No	No
Analysis						
Predicted m^6^A sites	Yes	No	Yes	Yes	Yes	Yes
Peak calling and annotations	Yes	Yes	Yes	Yes	Yes	Yes
Orthologous analysis	Yes	No	No	No	No	No
Co-methylation analysis	Yes	No	No	No	No	No
Post-transcription analysis	Yes	No	No	No	No	No
Blast to search RNA modification enzymes	Yes	No	No	No	No	No
Visualizations						
mRNA coordinate view	Yes	No	Yes	No	No	No
Genomic coordinate view	Yes	Yes	Yes	Yes	No	No
m^6^A visualizations	Yes	Yes	Yes	Yes	No	No
Gene-co-methylation Netviewer	Yes	No	No	No	No	No
Gene feature/types distribution	Yes	No	Yes	Yes	No	No
Coverage plots for different features	Yes	No	No	No	No	No
Boundary coverage for translation start/end and splice sites	Yes	No	No	No	No	No
mRNA metagene plot	Yes	No	No	Yes	No	No
Enriched motifs	Yes	No	No	Yes	Yes	Yes
Peak heatmaps near transcription/translation start and end sites	Yes	No	No	No	No	No
Gene Ontology functional enrichment for modified genes	Yes	No	No	No	No	No
Functional Pathway enrichment for modified genes	Yes	No	No	No	No	No
RNA characteristics of genes with modifications	Yes	No	No	No	No	No
The ways to query datasets						
Gene ID	Yes	Yes	Yes	Yes	Yes	Yes
Gene Name	Yes	No	Yes	Yes	No	No
Transcript ID	Yes	No	No	No	No	No
Sample accession	Yes	No	No	No	No	No
Study ID	Yes	No	No	No	No	No
PubMed ID	Yes	No	No	No	No	No
Links	http://bioinformatics.sc.cn/PRMD	http://rnamd.org/m6a/index.php	http://compgenomics.utsa.edu/MeTDB/	https://repicmod.uchicago.edu/repic	http://rna.sysu.edu.cn/rmbase	https://rna.sysu.edu.cn/encore/
Ref		(23)	(21)	(22)	(20)	

‘Other plant specific regulation datasets’ represents datasets from other relevant data sources. ‘Post-transcription analysis' contain the data analysis of alternative polyadenylation, exon usage, translational efficiency and differential expression. Most existed RNA modifications databases focus on mammal species without plants, especially human and mouse, such as m^6^Avar (74), RMVar (75) and WHISTLE (17). Compared with previous databases in the table which contain plant species, there has no database specifically covering RNA modification in multiple plant species.

Increasing numbers of recent studies in diverse plant species have profiled several RNA modifications in different tissues, developmental stages and stress conditions, which may be relevant for constructing an integrated plant RNA modification database. We developed PRMD by collecting and processing MeRIP-seq data generated in previous studies on 20 plant species. Moreover, we incorporated several convenient tools, such as RMlevelDiff, RMplantVar, RNAmodNet and Blast (for data analyses) as well as JBrowse, mRNAbrowse, RNAlollipop and IGV (for visualizing diverse datasets). Additionally, PRMD provides information regarding annotations and may be used to analyze APA, exon usage, TE and differential expression. Moreover, PRMD intuitively displays the related datasets from previous studies and other resources (e.g. eQTLs, SNVs, GWAS, sORFs, RNA loops, rG4 structures, RBP binding sites, RNA secondary structures and APAs).

## Perspectives

There is increasing evidence of the links between aberrant RNA modifications and many key biological processes in plants. In particular, m^6^A, which is the most prevalent and abundant RNA modification, reversibly regulates RNA processing and metabolism related to plant development, evolution and pathological processes. Although several databases have been constructed to integrate the existing sequencing datasets and different types of RNA modifications, a comprehensive and easy-to-use database comprising information regarding diverse RNA modifications in plant species is still lacking. Therefore, we developed PRMD primarily to facilitate research on plant RNA modifications. Currently, PRMD includes six RNA modification types in 20 plant species. One limitation of this database is that there are relatively few MeRIP-seq samples for several of the plant species (5 of 20) and relevant datasets were not included for all species. For example, eQTL datasets are available for only *A. thaliana* and *O. sativa*, while R-loop datasets are available for only *A. thaliana*, *G. max*, *O. sativa* and *Z. mays*. Because of the continual development of high-throughput sequencing technology, there will likely be a rapid increase in the number of available sequencing datasets, which will greatly enhance research on RNA modifications. We will regularly update PRMD and add sequencing and related datasets to cover more plant species and RNA modifications. Furthermore, PRMD includes several convenient visualization and functional analysis tools. As a long-term research project, we will develop more helpful tools for functional analyses of PRMD datasets. We believe that PRMD is a very convenient and comprehensive database of plant RNA modifications.

## Supplementary Material

gkad851_Supplemental_File

## Data Availability

PRMD is a comprehensive and convenient interactive online database available at http://bioinformatics.sc.cn/PRMD/ and http://rnainformatics.org.cn/PRMD. The raw-sequencing MeRIP-seq data of *Oryza rufipogon* and two cultivated rice subspecies have been deposited in the Genome Sequence Archive (GSA) database of the National Genomics Data Center (NGDC; https://ngdc.cncb.ac.cn/gsa/), which can be obtained via BioProject accession PRJCA018161. The pipeline code for Merip-seq data processing used in this study have been deposited in github (https://github.com/rnainformatics/PRMD) and figshare (https://doi.org/10.6084/m9.figshare.24139029).
