# Supplementary material for: PRMD: an integrated database for plant RNA modifications
Source: Nucleic Acids Res. 2023 Oct 13;52(D1):D1597–613. doi: 10.1093/nar/gkad851 (PMC10768107; doi:10.1093/nar/gkad851)
Supplement: gkad851_Supplemental_File [file gkad851_supplemental_file.pdf]

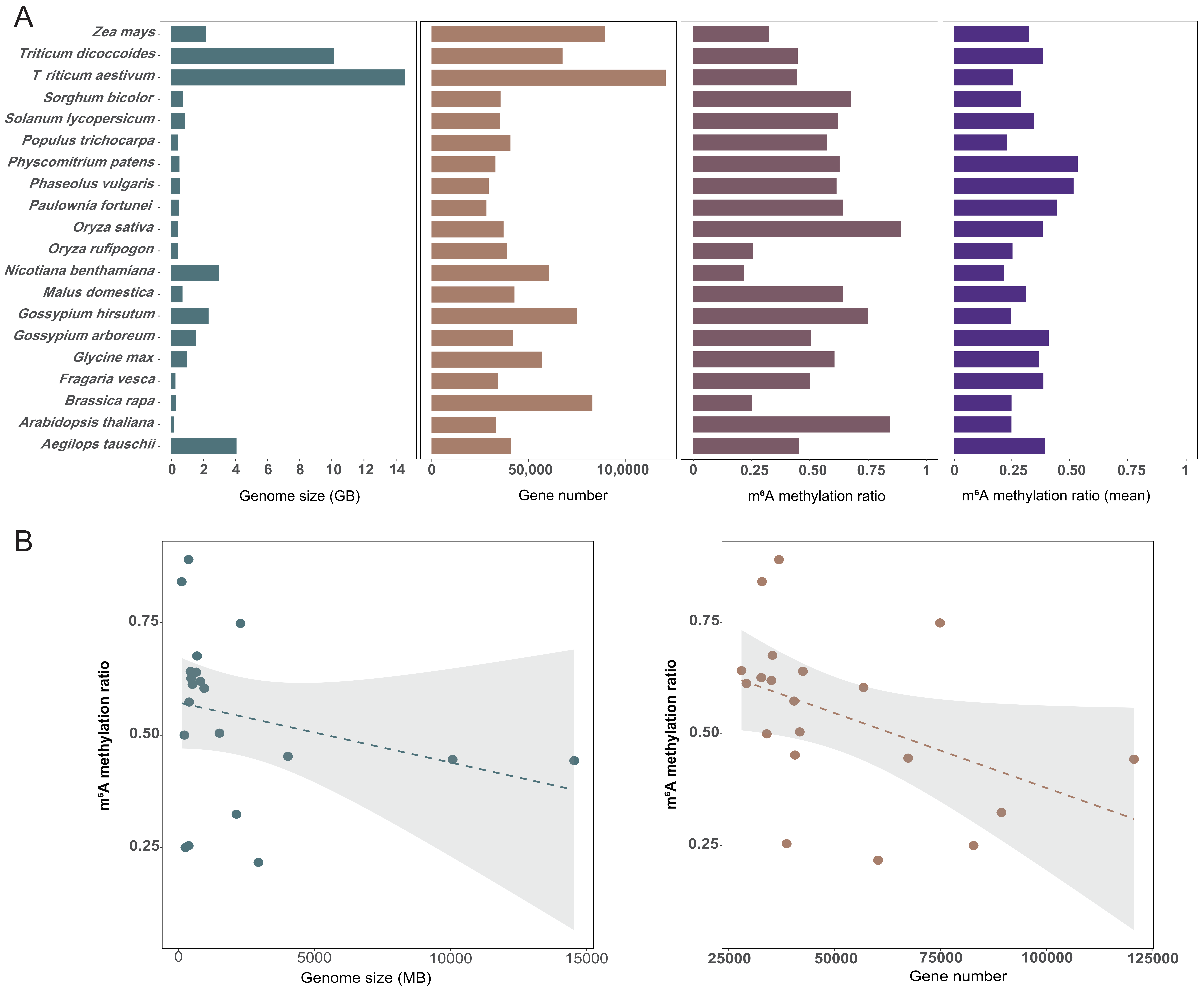

Figure S1. Characterization of m^{6}A methylomes in 20 plant species. (A) Overview of the genome size, gene number, transcriptome-wide m^{6}A methylation ratio and mean m6A methylation ratio among the samples collected for 20 plant species. (B) The m^{6}A methylation ratio was negatively correlated with genome size and gene number.

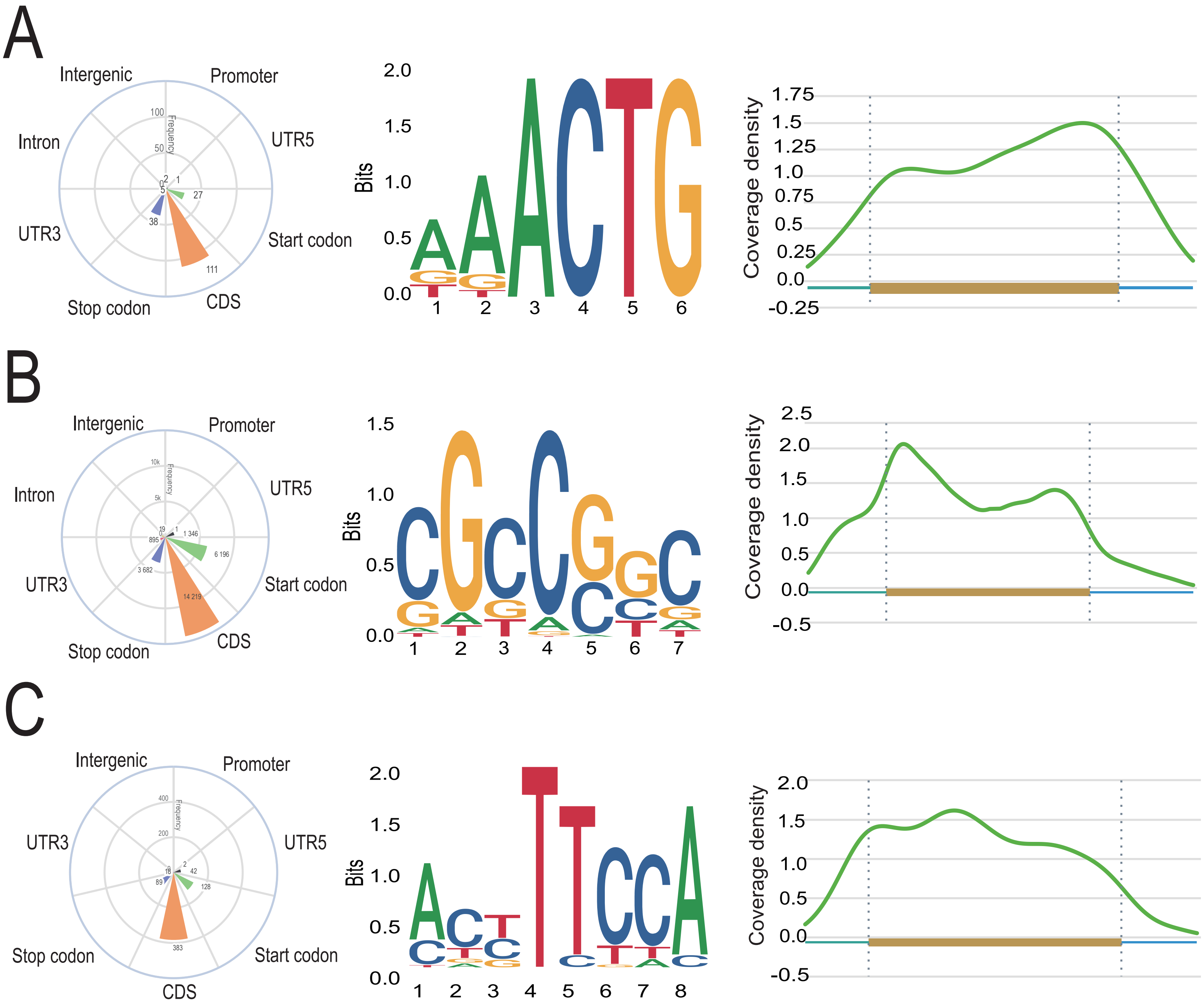

Figure S2. Distributions of m^{6}A, m^{5}C and pseudouridine. (A–C) Coverage plot, enriched motif plot and metagene plot of the m^{6}A, m^{5}C and pseudouridine sites.

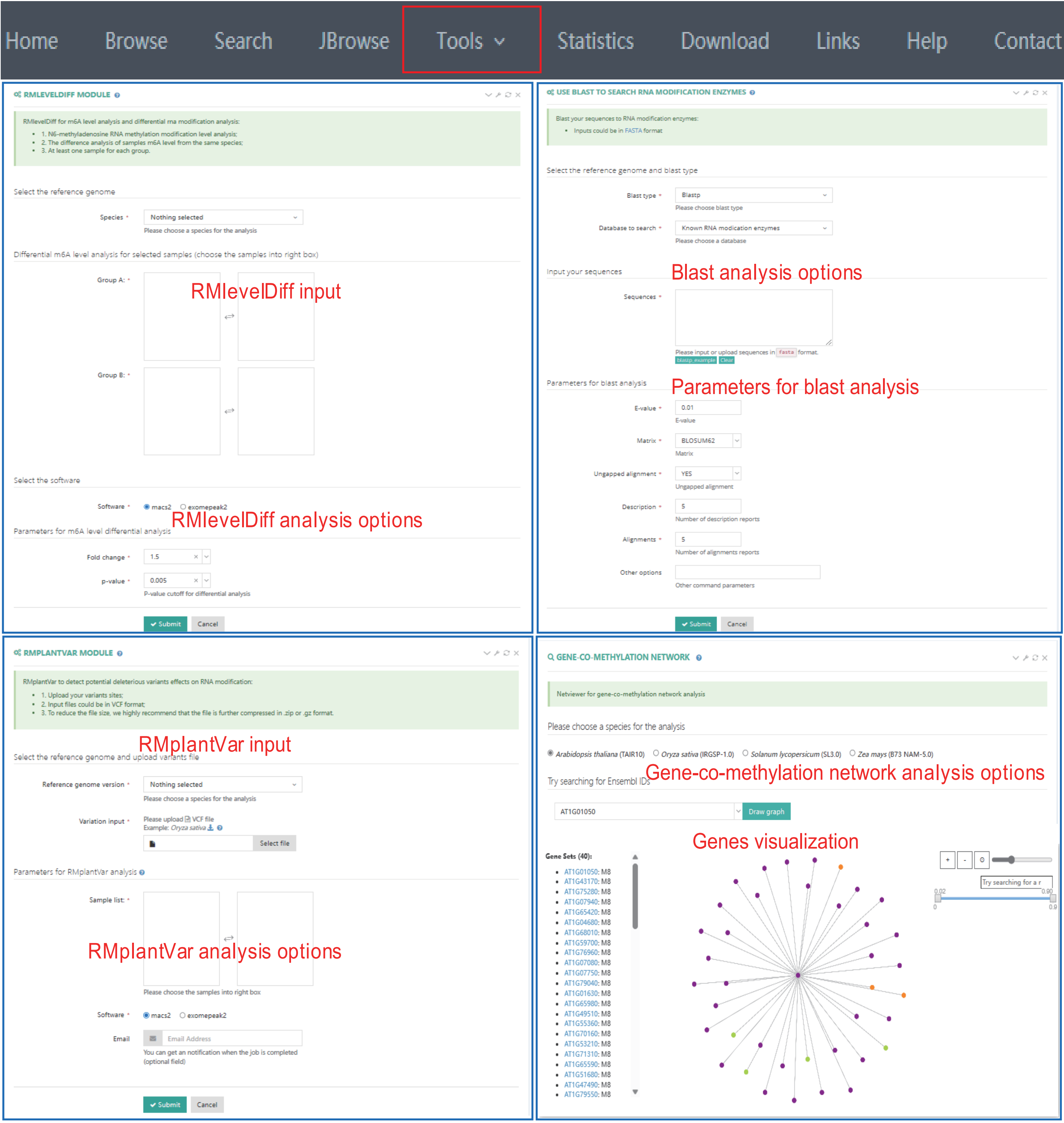

Figure S3. Screenshots of four useful tools in PRMD. RMlevelDiff parameters for analyzing RNA modification levels and differential modifications. RMplantVar was used for detecting potential deleterious variant effects on RNA modifications. RNAmodNet was used for gene co-methylation network analyses and the visualization of the m^6A co-modification gene network, while Blast was used for identifying potential RNA modification-related enzymes among the sequences provided by users..

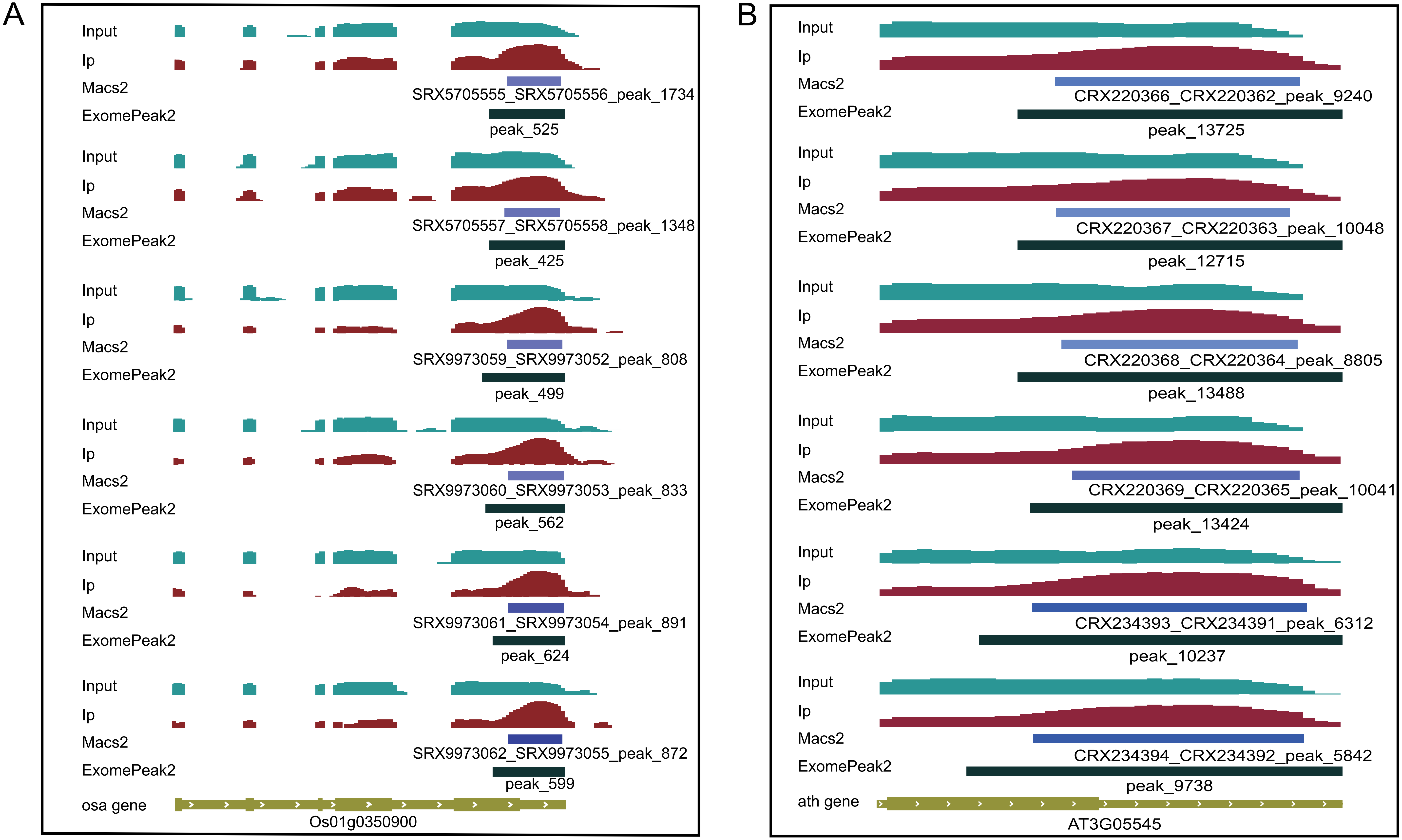

Figure S4. Visualization of the IGV data for the association between the agronomic trait-related gene IPI1 in *Oryza sativa* (Os01g0350900) and its orthologous gene in *Arabidopsis thaliana* (AT3G05545).

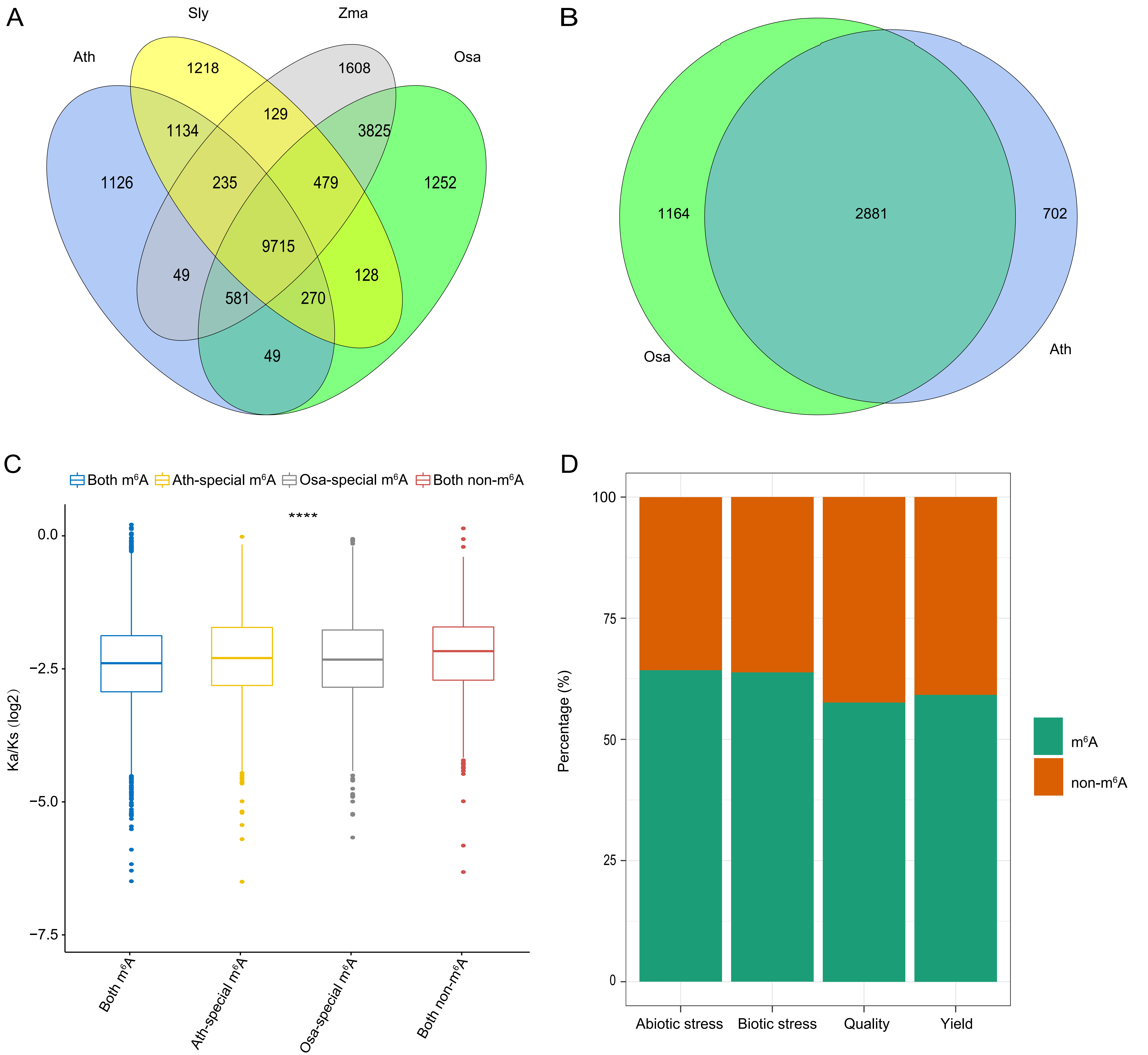

Figure S5. Orthogroup gene identification and evolution analyses. (A) Venn diagram of 1,126 (*Arabidopsis thaliana*), 1,252 (*Oryza sativa*), 1,218 (*Solanum lycopersicum*), 1,608 (*Zea mays*) species-specific orthogroups and 9,715 common orthogroups. (B) One-to-one orthologous gene pairs with m^6A modifications between *A. thaliana* and *O. sativa*. (C) Comparative analysis of the evolution of one-to-one orthologous m^6A-modified gene pairs between *A. thaliana* and *O. sativa*. (D) Proportion of m^6A-modified genes that are associated with agronomic traits.

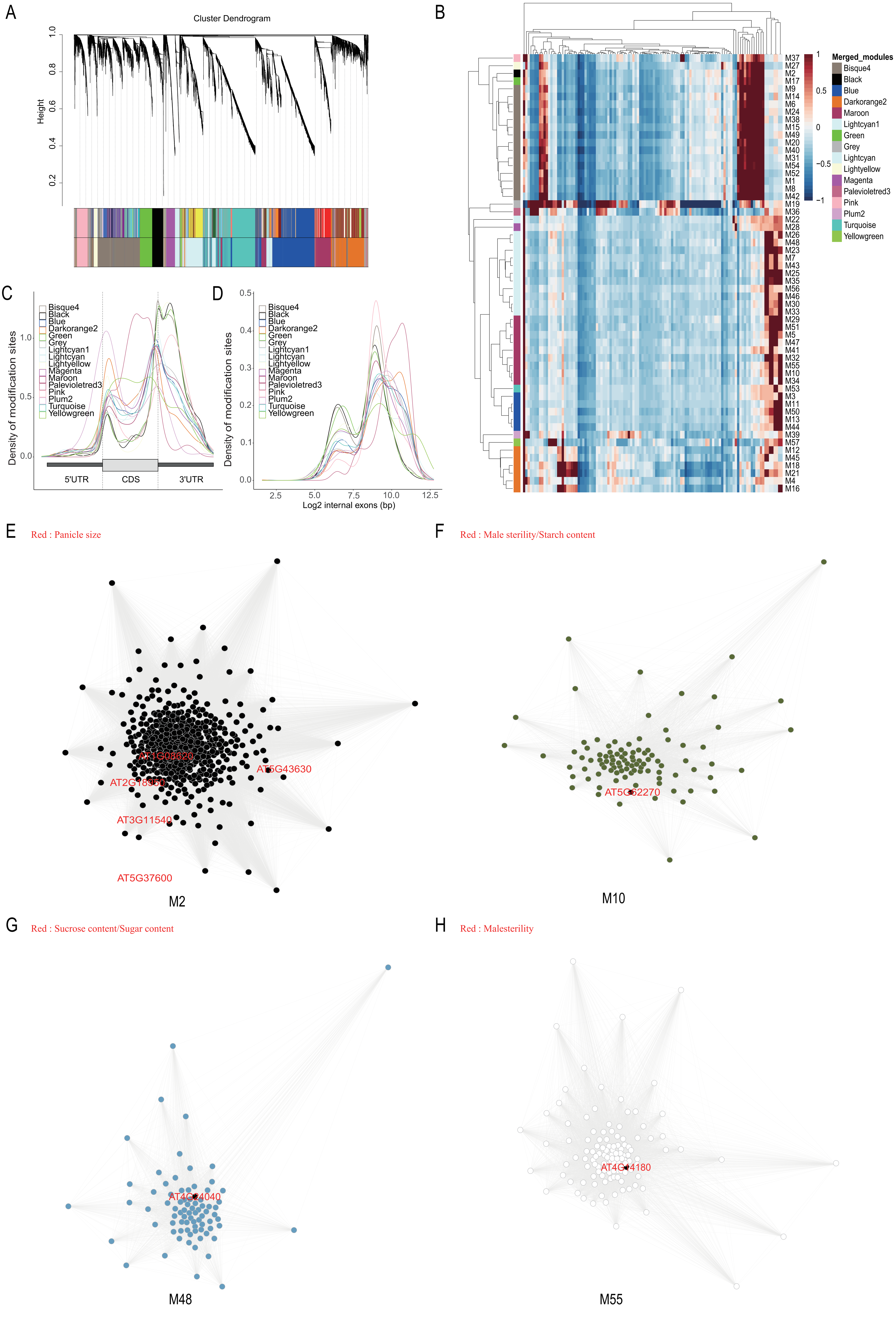

Figure S6. Classification and analyses of co-methylated m^{6}A modules in *Arabidopsis thaliana.* (A) Co-methylated m^{6}A modules. (B) Heatmap presenting the m^{6}A indices of all co-methylation modules across all *A. thaliana* samples. (C) Density distributions of m^{6}A peaks in different modules across the 5′ UTR, CDS and 3′ UTR. (D) Density distributions of the log-transformed lengths of the internal exons with m^{6}A peaks in different combined co-methylation modules. (E-H) In *A. thaliana*, modules M2 (E), M10 (F), M48 (G) and M55 (H) were associated with panicle size, male sterility/starch content, sucrose content/sugar content and male sterility, respectively.

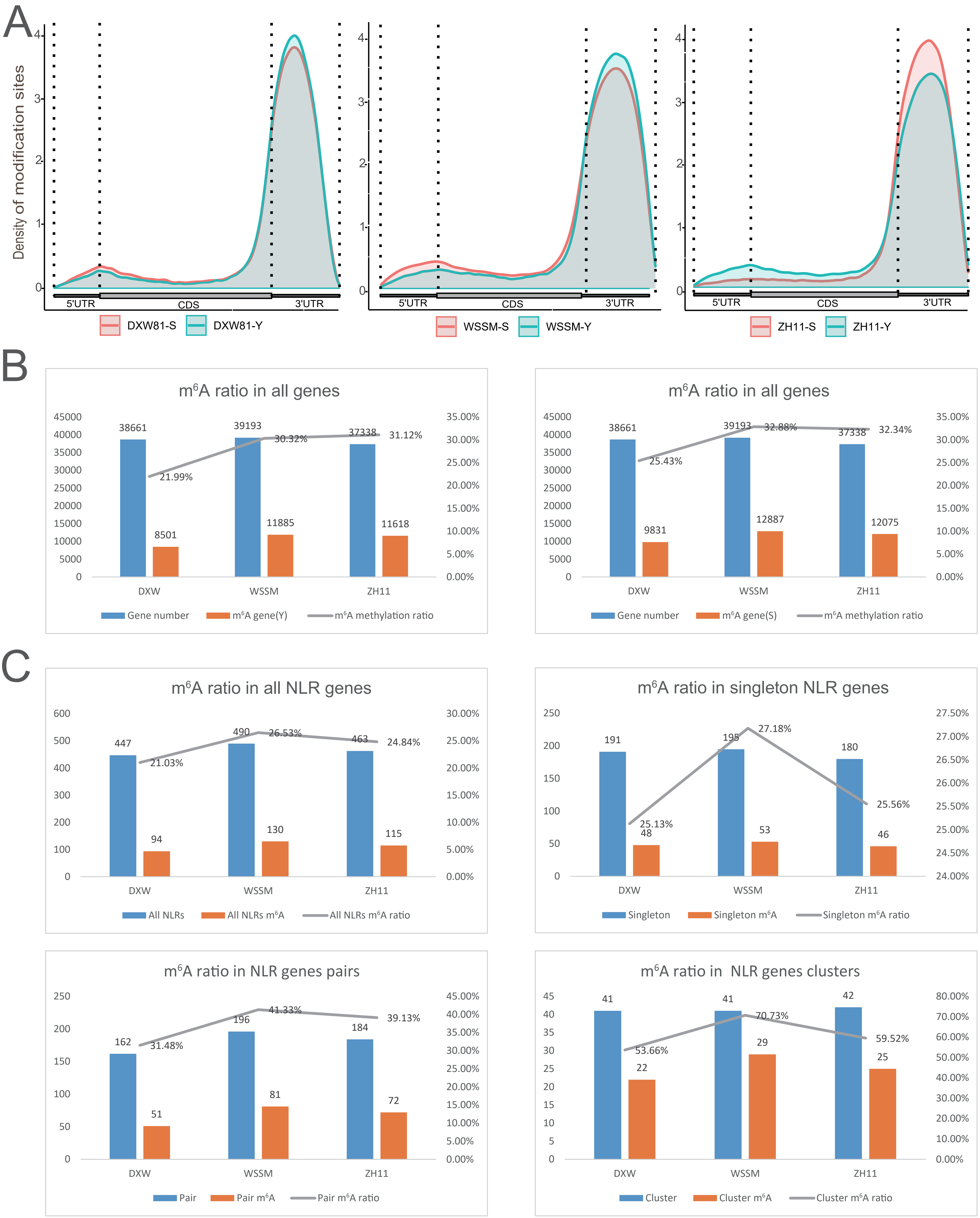

Figure S7. Comparative analysis of wild rice (Oryza rufipogon) and two cultivated rice subspecies (*Oryza sativa ssp. indica* and *Oryza sativa ssp. japonica*). (A) Density distributions of m6A peaks across the 5′ UTR, CDS and 3′ UTR. (B) m^{6}A methylation ratio of all genes in O. rufipogon, O. sativa ssp. indica and O. sativa ssp. japonica. (C) m^{6}A methylation ratio of singleton genes, gene pairs and gene clusters (NLR gene).

Table S1. MeRIP-Seq datasets information in PRMD.

Organism	Ip	Input	Study	CreateDate	Macs2 peaks count	ExomePeak2 peaks count	Macs2 gene count	ExomePeak2 gene count	PMID
Aegilops tauschii	CRX237813	CRX237812	CRA004114	2021/4/27	21844	31957	13866	18112	34633447
Arabidopsis thaliana	CRX121097	CRX121096	CRA002826	2020/6/19	6697	22214	6529	12950	29180595
Arabidopsis thaliana	CRX121099	CRX121098	CRA002826	2020/6/19	7564	23821	7161	13918	29180595
Arabidopsis thaliana	CRX121101	CRX121100	CRA002826	2020/6/19	5704	25319	5470	13516	29180595
Arabidopsis thaliana	CRX121103	CRX121102	CRA002826	2020/6/19	5195	24835	5077	13142	29180595
Arabidopsis thaliana	CRX220366	CRX220362	CRA003884	2021/2/3	21643	32259	15989	18680	34178005
Arabidopsis thaliana	CRX220367	CRX220363	CRA003884	2021/2/3	23098	29815	15823	18103	34178005
Arabidopsis thaliana	CRX220368	CRX220364	CRA003884	2021/2/3	20491	31578	15768	18807	34178005
Arabidopsis thaliana	CRX220369	CRX220365	CRA003884	2021/2/3	23332	31506	16247	18829	34178005
Arabidopsis thaliana	CRX238892	CRX238891	CRA003884	2021/2/3	14055	25007	12697	16756	34178005
Arabidopsis thaliana	CRX234393	CRX234391	CRA004052	2022/1/6	14740	23977	13905	17816	NA
Arabidopsis thaliana	CRX234394	CRX234392	CRA004052	2022/1/6	13622	22967	12830	17532	NA
Arabidopsis thaliana	CRX235013	CRX235011	CRA004052	2022/1/6	13414	22264	12929	17222	NA
Arabidopsis thaliana	CRX235014	CRX235012	CRA004052	2022/1/6	13065	20778	12946	17075	NA
Arabidopsis thaliana	CRX292819	CRX292818	CRA004052	2022/1/6	13192	26426	12765	18282	NA
Arabidopsis thaliana	CRX292821	CRX292820	CRA004052	2022/1/6	13741	27548	13013	18716	NA
Arabidopsis thaliana	CRX237799	CRX237798	CRA004114	2021/4/27	13528	24097	12524	18256	34633447
Arabidopsis thaliana	SRX647392	SRX647391	SRP044116	2015/7/22	6392	11525	5830	8747	25430002
Arabidopsis thaliana	SRX647394	SRX647393	SRP044116	2015/7/22	4889	10783	4653	8351	25430002
Arabidopsis thaliana	SRX647396	SRX647395	SRP044116	2015/7/22	5213	13499	4954	9917	25430002
Arabidopsis thaliana	SRX647398	SRX647397	SRP044116	2015/7/22	4251	12547	4101	9193	25430002
Arabidopsis thaliana	SRX1456205	SRX1456204	SRP066804	2016/7/25	3424	380	3484	371	27396363
Arabidopsis thaliana	SRX1456207	SRX1456206	SRP066804	2016/7/25	2890	302	2952	300	27396363

Arabidopsis thaliana	SRX1456209	SRX1456208	SRP066804	2016/7/25	2236	139	1385	104	27396363
Arabidopsis thaliana	SRX1456211	SRX1456210	SRP066804	2016/7/25	1558	193	916	156	27396363
Arabidopsis thaliana	SRX1656426	SRX1656425	SRP072217	2018/2/8	6697	22214	6529	12950	29180595
Arabidopsis thaliana	SRX1656428	SRX1656427	SRP072217	2018/2/8	7564	23821	7161	13918	29180595
Arabidopsis thaliana	SRX1656430	SRX1656429	SRP072217	2018/2/8	5704	25319	5470	13516	29180595
Arabidopsis thaliana	SRX1656432	SRX1656431	SRP072217	2018/2/8	5195	24835	5077	13142	29180595
Arabidopsis thaliana	SRX3532630	SRX3532629	SRP128056	2018/11/9	6774	8507	6916	7167	30380407; 32817553
Arabidopsis thaliana	SRX3532632	SRX3532631	SRP128056	2018/11/9	4821	4418	5019	3936	30380407; 32817553
Arabidopsis thaliana	SRX3532634	SRX3532633	SRP128056	2018/11/9	789	1123	683	992	30380407; 32817553
Arabidopsis thaliana	SRX3532636	SRX3532635	SRP128056	2018/11/9	376	768	384	646	30380407; 32817553
Arabidopsis thaliana	SRX4110180	SRX4110179	SRP148623	2018/5/22	83	154	27	113	NA
Arabidopsis thaliana	SRX4312802	SRX4312806	SRP151450	2018/11/9	8299	8000	8679	7228	30380407; 32817553
Arabidopsis thaliana	SRX4312803	SRX4312807	SRP151450	2018/11/9	8333	8300	8799	7624	30380407; 32817553
Arabidopsis thaliana	SRX4312804	SRX4312808	SRP151450	2018/11/9	10059	10574	10429	9316	30380407; 32817553
Arabidopsis thaliana	SRX4312805	SRX4312809	SRP151450	2018/11/9	9704	9760	10297	8887	30380407; 32817553
Arabidopsis thaliana	SRX5007196	SRX5007195	SRP168611	2020/9/21	1111	35758	999	14773	32817553
Arabidopsis thaliana	SRX5007198	SRX5007197	SRP168611	2020/9/21	1426	35747	839	17247	32817553
Arabidopsis thaliana	SRX10913494	SRX10913493	SRP320161	2022/1/21	3420	11338	3517	8502	35236848

Arabidopsis thaliana	SRX10913496	SRX10913495	SRP320161	2022/1/21	10977	20918	10839	14865	35236848
Arabidopsis thaliana	SRX10913498	SRX10913497	SRP320161	2022/1/21	10446	23611	9947	15210	35236848
Arabidopsis thaliana	SRX10913500	SRX10913499	SRP320161	2022/1/21	4752	8571	4202	6635	35236848
Arabidopsis thaliana	SRX10913502	SRX10913501	SRP320161	2022/1/21	7099	14363	6053	10107	35236848
Arabidopsis thaliana	SRX10913504	SRX10913503	SRP320161	2022/1/21	9096	7743	4398	6073	35236848
Arabidopsis thaliana	SRX10913506	SRX10913505	SRP320161	2022/1/21	1781	14123	1353	9345	35236848
Arabidopsis thaliana	SRX10913508	SRX10913507	SRP320161	2022/1/21	1152	10000	813	6695	35236848
Arabidopsis thaliana	SRX10913510	SRX10913509	SRP320161	2022/1/21	4503	16857	3882	10546	35236848
Arabidopsis thaliana	SRX10913512	SRX10913511	SRP320161	2022/1/21	8921	23144	8360	14217	35236848
Arabidopsis thaliana	SRX10913514	SRX10913513	SRP320161	2022/1/21	6153	20660	5878	12761	35236848
Arabidopsis thaliana	SRX10913516	SRX10913515	SRP320161	2022/1/21	8772	23463	8474	14538	35236848
Arabidopsis thaliana	SRX11542396	SRX11542402	SRP329693	2021/8/9	11085	20257	10704	15747	NA
Arabidopsis thaliana	SRX11542397	SRX11542403	SRP329693	2021/8/9	11278	20665	10893	16118	NA
Arabidopsis thaliana	SRX11542398	SRX11542404	SRP329693	2021/8/9	11475	21082	10997	16304	NA
Arabidopsis thaliana	SRX11542399	SRX11542405	SRP329693	2021/8/9	11459	21297	10916	16208	NA
Arabidopsis thaliana	SRX11542400	SRX11542406	SRP329693	2021/8/9	12086	21454	11495	16641	NA
Arabidopsis thaliana	SRX11542401	SRX11542407	SRP329693	2021/8/9	11359	20645	11047	16461	NA
Arabidopsis thaliana	SRX1182476	SRX1182482	SRP063314	2015/10/28	2403	581	2515	506	29131848; 26667818
Arabidopsis thaliana	SRX1182477	SRX1182483	SRP063314	2015/10/28	5611	3846	5712	3767	29131848; 26667818
Arabidopsis thaliana	SRX1182478	SRX1182484	SRP063314	2015/10/28	4724	3128	4824	3038	29131848; 26667818
Arabidopsis thaliana	SRX1182485	SRX1182491	SRP063314	2015/10/28	270	190	246	97	29131848; 26667818

Arabidopsis thaliana	SRX1182486	SRX1182492	SRP063314	2015/10/28	981	682	946	676	29131848; 26667818
Arabidopsis thaliana	SRX1182487	SRX1182493	SRP063314	2015/10/28	811	932	803	881	29131848; 26667818
Arabidopsis thaliana	SRX12172342	SRX12172348	SRP336880	2022/08/01	7806	13541	7124	10634	35710867
Arabidopsis thaliana	SRX12172343	SRX12172349	SRP336880	2022/08/01	7195	12888	6400	9881	35710867
Arabidopsis thaliana	SRX12172344	SRX12172350	SRP336880	2022/08/01	3185	5969	3145	5291	35710867
Arabidopsis thaliana	SRX12172345	SRX12172351	SRP336880	2022/08/01	6127	11173	5737	8889	35710867
Arabidopsis thaliana	SRX12172346	SRX12172352	SRP336880	2022/08/01	7940	13225	7251	10411	35710867
Arabidopsis thaliana	SRX12172347	SRX12172353	SRP336880	2022/08/01	7484	13351	6995	10747	35710867
Arabidopsis thaliana	SRX13571648	SRX13571650	SRP353075	2022/8/29	5635	22313	5577	15693	NA
Arabidopsis thaliana	SRX13571651	SRX13571652	SRP353075	2022/8/29	6587	22620	6399	15647	NA
Arabidopsis thaliana	SRX13571653	SRX13571670	SRP353075	2022/8/29	9033	24663	8355	16246	NA
Arabidopsis thaliana	SRX13571654	SRX13571655	SRP353075	2022/8/29	7988	24923	7418	15780	NA
Arabidopsis thaliana	SRX13571656	SRX13571657	SRP353075	2022/8/29	6542	22811	6320	15024	NA
Arabidopsis thaliana	SRX13571658	SRX13571659	SRP353075	2022/8/29	6378	24247	6313	17085	NA
Arabidopsis thaliana	SRX13571660	SRX13571661	SRP353075	2022/8/29	6701	23673	6812	17517	NA
Arabidopsis thaliana	SRX13571662	SRX13571663	SRP353075	2022/8/29	5768	22981	5815	16499	NA
Arabidopsis thaliana	SRX13571664	SRX13571665	SRP353075	2022/8/29	8749	26464	8489	18154	NA
Arabidopsis thaliana	SRX13571673	SRX13571674	SRP353075	2022/8/29	7095	23577	6754	15744	NA
Arabidopsis thaliana	SRX13571675	SRX13571676	SRP353075	2022/8/29	5656	22567	4830	14007	NA
Arabidopsis thaliana	SRX13571677	SRX13571678	SRP353075	2022/8/29	6676	26658	5433	15123	NA
Arabidopsis thaliana	SRX13571679	SRX13571680	SRP353075	2022/8/29	7177	24578	6010	14994	NA
Arabidopsis thaliana	SRX13571681	SRX13571682	SRP353075	2022/8/29	7979	28826	6343	15870	NA
Arabidopsis thaliana	SRX13571683	SRX13571684	SRP353075	2022/8/29	6244	21211	6068	14738	NA
Arabidopsis thaliana	SRX13571685	SRX13571686	SRP353075	2022/8/29	6608	22480	6356	15458	NA

Arabidopsis thaliana	SRX18210098	SRX18210112	SRP406884	2022/11/8	11149	22070	9288	12135	36460653
Arabidopsis thaliana	SRX18210099	SRX18210113	SRP406884	2022/11/8	10124	19374	8658	11157	36460653
Arabidopsis thaliana	SRX18210106	SRX18210100	SRP406884	2022/11/8	10929	21894	9123	12037	36460653
Arabidopsis thaliana	SRX18210107	SRX18210101	SRP406884	2022/11/8	11389	22348	9523	12465	36460653
Arabidopsis thaliana	SRX18210108	SRX18210102	SRP406884	2022/11/8	9206	19655	7011	9650	36460653
Arabidopsis thaliana	SRX18210109	SRX18210103	SRP406884	2022/11/8	8038	17439	6110	8593	36460653
Arabidopsis thaliana	SRX18210110	SRX18210104	SRP406884	2022/11/8	11502	21619	8374	10690	36460653
Arabidopsis thaliana	SRX18210111	SRX18210105	SRP406884	2022/11/8	10637	21584	7761	10435	36460653
Arabidopsis thaliana	SRX5322276	SRX5322279	SRP183144	2022/2/2	970	1576	812	964	32817553
Arabidopsis thaliana	SRX5322278	SRX5322277	SRP183144	2022/2/2	18	318	21	266	32817553
Arabidopsis thaliana	SRX7567705	SRX7567715	SRP242058	2022/1/16	12160	21182	10755	14761	NA
Arabidopsis thaliana	SRX7567706	SRX7567716	SRP242058	2022/1/16	13444	20937	12566	15923	NA
Arabidopsis thaliana	SRX8151465	SRX8151464	SRP257780	2020/4/23	737	3322	131	2638	NA
Arabidopsis thaliana	SRX8151466	SRX8151464	SRP257780	2020/4/23	503	2594	117	2057	NA
Arabidopsis thaliana	SRX8840862	SRX8840859	SRP274017	2021/7/17	1176	172	1149	167	34650267
Arabidopsis thaliana	SRX8840863	SRX8840860	SRP274017	2021/7/17	1706	326	1705	300	34650267
Arabidopsis thaliana	SRX8840864	SRX8840861	SRP274017	2021/7/17	1584	155	1551	145	34650267
Arabidopsis thaliana	SRX8840868	SRX8840865	SRP274017	2021/7/17	2789	1067	2910	1047	34650267
Arabidopsis thaliana	SRX8840869	SRX8840866	SRP274017	2021/7/17	3203	1639	3315	1594	34650267
Arabidopsis thaliana	SRX8840870	SRX8840867	SRP274017	2021/7/17	2773	1063	2867	1029	34650267
Arabidopsis thaliana	SRX8840874	SRX8840871	SRP274017	2021/7/17	2943	604	2992	575	34650267
Arabidopsis thaliana	SRX8840875	SRX8840872	SRP274017	2021/7/17	3253	1281	3314	1244	34650267
Arabidopsis thaliana	SRX8840876	SRX8840873	SRP274017	2021/7/17	3503	762	3509	730	34650267
Arabidopsis thaliana	SRX8840880	SRX8840877	SRP274017	2021/7/17	1955	605	2032	585	34650267
Arabidopsis thaliana	SRX8840881	SRX8840878	SRP274017	2021/7/17	2442	811	2546	788	34650267
Arabidopsis thaliana	SRX8840882	SRX8840879	SRP274017	2021/7/17	1955	546	2021	516	34650267

Arabidopsis thaliana	SRX9910817	SRX9910816	SRP248245	2021/3/16	9503	12242	7937	7884	33515769
Arabidopsis thaliana	SRX9910819	SRX9910818	SRP248245	2021/3/16	11469	13742	8563	7971	33515769
Arabidopsis thaliana	SRX9910821	SRX9910820	SRP248245	2021/3/16	8564	11875	7320	7647	33515769
Brassica rapa	SRX8783095	SRX8783094	SRP272790	2021/8/31	4839	16686	3989	11867	NA
Brassica rapa	SRX8783097	SRX8783096	SRP272790	2021/8/31	4785	18529	3869	12934	NA
Brassica rapa	SRX8783099	SRX8783098	SRP272790	2021/8/31	5029	16916	4121	12262	NA
Brassica rapa	SRX8783101	SRX8783100	SRP272790	2021/8/31	6149	20998	4931	14430	NA
Brassica rapa	SRX8783103	SRX8783102	SRP272790	2021/8/31	4984	21776	3937	14404	NA
Brassica rapa	SRX8783105	SRX8783104	SRP272790	2021/8/31	996	25451	823	14670	NA
Brassica rapa	SRX8724844	SRX8724843	SRP271711	2020/7/16	3558	11378	3003	8516	32842619
Brassica rapa	SRX8724846	SRX8724845	SRP271711	2020/7/16	3474	13566	2863	9939	32842619
Brassica rapa	SRX8724848	SRX8724847	SRP271711	2020/7/16	3905	13780	3257	10235	32842619
Brassica rapa	SRX8724850	SRX8724849	SRP271711	2020/7/16	5162	15554	4127	11307	32842619
Brassica rapa	SRX8724852	SRX8724851	SRP271711	2020/7/16	4712	16103	3725	11293	32842619
Brassica rapa	SRX8724854	SRX8724853	SRP271711	2020/7/16	733	18879	613	11906	32842619
Fragaria vesca	SRX10145172	SRX10145175	SRP307368	2021/5/18	14507	21395	12388	14710	34078442
Fragaria vesca	SRX10145173	SRX10145176	SRP307368	2021/5/18	14679	20961	12567	14830	34078442
Fragaria vesca	SRX10145174	SRX10145177	SRP307368	2021/5/18	14008	21109	12239	14752	34078442
Fragaria vesca	SRX10145178	SRX10145181	SRP307368	2021/5/18	15970	22193	12334	14030	34078442
Fragaria vesca	SRX10145179	SRX10145182	SRP307368	2021/5/18	16095	23369	12633	14761	34078442
Fragaria vesca	SRX10145180	SRX10145183	SRP307368	2021/5/18	16206	23140	12504	14551	34078442
Fragaria vesca	SRX10145184	SRX10145187	SRP307368	2021/5/18	14652	19399	11664	12850	34078442
Fragaria vesca	SRX10145185	SRX10145188	SRP307368	2021/5/18	14744	20418	11584	13157	34078442
Fragaria vesca	SRX10145186	SRX10145189	SRP307368	2021/5/18	15069	20239	11752	13126	34078442
Gossypium arboreum	CRX237801	CRX237800	CRA004114	2021/4/27	20660	27617	13382	20764	34633447
Gossypium hirsutum	CRX237803	CRX237802	CRA004114	2021/4/27	38800	58198	28576	43612	34633447

Gossypium hirsutum	SRX9196417	SRX9196424	SRP285473	2021/12/31	4299	55983	490	31443	DOI:10.1016/j.envexpbot.2021.104729
Gossypium hirsutum	SRX9196418	SRX9196425	SRP285473	2021/12/31	5001	53642	631	30467	DOI:10.1016/j.envexpbot.2021.104729
Gossypium hirsutum	SRX9196420	SRX9196415	SRP285473	2021/12/31	1068	46853	167	27726	DOI:10.1016/j.envexpbot.2021.104729
Gossypium hirsutum	SRX9196421	SRX9196416	SRP285473	2021/12/31	1994	45200	280	26808	DOI:10.1016/j.envexpbot.2021.104729
Gossypium hirsutum	SRX9196422	SRX9196419	SRP285473	2021/12/31	9375	65215	873	35045	DOI:10.1016/j.envexpbot.2021.104729
Gossypium hirsutum	SRX9196426	SRX9196423	SRP285473	2021/12/31	3524	58276	344	32587	DOI:10.1016/j.envexpbot.2021.104729
Glycine max	CRX237807	CRX237806	CRA004114	2021/4/27	29700	45131	23316	31843	34633447
Glycine max	SRX13770378	SRX13770377	SRP354877	2022/10/14	18865	30741	16307	24253	36113351

Glycine max	SRX13770380	SRX13770379	SRP354877	2022/10/14	18531	29949	16059	23906	36113351
Glycine max	SRX13770382	SRX13770381	SRP354877	2022/10/14	18712	30710	16041	24102	36113351
Glycine max	SRX13770384	SRX13770383	SRP354877	2022/10/14	18916	30937	16166	24227	36113351
Glycine max	SRX13770386	SRX13770385	SRP354877	2022/10/14	18956	31068	16192	24312	36113351
Glycine max	SRX13770388	SRX13770387	SRP354877	2022/10/14	18853	30687	16270	24272	36113351
Glycine max	SRX13770390	SRX13770389	SRP354877	2022/10/14	18791	30223	16226	24135	36113351
Glycine max	SRX13770392	SRX13770391	SRP354877	2022/10/14	18293	30213	15947	23920	36113351
Glycine max	SRX13770394	SRX13770393	SRP354877	2022/10/14	18164	30295	15854	23998	36113351
Malus domestica	SRX12683309	SRX12683322	SRP342071	2022/10/1	13728	16470	9331	12544	35246985
Malus domestica	SRX12683310	SRX12683323	SRP342071	2022/10/1	13686	16590	9636	12658	35246985
Malus domestica	SRX12683311	SRX12683324	SRP342071	2022/10/1	13389	16490	9192	12475	35246985
Malus domestica	SRX12683312	SRX12683325	SRP342071	2022/10/1	13084	15600	8948	11746	35246985
Malus domestica	SRX12683313	SRX12683326	SRP342071	2022/10/1	14112	17068	9792	12761	35246985
Malus domestica	SRX12683314	SRX12683327	SRP342071	2022/10/1	12873	17073	9127	12757	35246985
Malus domestica	SRX12683315	SRX12683329	SRP342071	2022/10/1	12785	16161	9034	12006	35246985
Malus domestica	SRX12683316	SRX12683330	SRP342071	2022/10/1	13191	17848	9302	13066	35246985
Malus domestica	SRX12683318	SRX12683331	SRP342071	2022/10/1	13720	17301	9390	12796	35246985
Malus domestica	SRX12683319	SRX12683332	SRP342071	2022/10/1	12176	15155	8635	11381	35246985
Malus domestica	SRX12683320	SRX12683333	SRP342071	2022/10/1	11179	15001	8357	11327	35246985
Malus domestica	SRX12683321	SRX12683334	SRP342071	2022/10/1	12145	15630	8538	11648	35246985
Malus domestica	SRX13198177	SRX13198180	SRP347250	2021/12/29	23147	28129	15873	20251	35009106
Malus domestica	SRX13198178	SRX13198180	SRP347250	2021/12/29	26722	27643	16633	20114	35009106
Malus domestica	SRX13198185	SRX13198179	SRP347250	2021/12/29	24845	26383	17141	19818	35009106
Malus domestica	SRX13198186	SRX13198179	SRP347250	2021/12/29	24706	26569	17475	19955	35009106
Malus domestica	SRX13198187	SRX13198179	SRP347250	2021/12/29	26006	25945	17598	19624	35009106
Malus domestica	SRX13198188	SRX13198180	SRP347250	2021/12/29	26625	28022	16634	20881	35009106

Nicotiana benthamiana	SRX14619751	SRX14619768	SRP365980	2022/10/9	2234	7458	1493	5373	35700092
Nicotiana benthamiana	SRX14619752	SRX14619753	SRP365980	2022/10/9	2406	6475	1584	4681	35700092
Nicotiana benthamiana	SRX14619761	SRX14619754	SRP365980	2022/10/9	2357	6188	1554	4548	35700092
Nicotiana benthamiana	SRX14619762	SRX14619755	SRP365980	2022/10/9	1327	4493	939	3282	35700092
Nicotiana benthamiana	SRX14619763	SRX14619756	SRP365980	2022/10/9	1370	4224	1009	3116	35700092
Nicotiana benthamiana	SRX14619764	SRX14619757	SRP365980	2022/10/9	1350	4272	930	3140	35700092
Nicotiana benthamiana	SRX14619765	SRX14619758	SRP365980	2022/10/9	2137	6822	1333	4960	35700092
Nicotiana benthamiana	SRX14619766	SRX14619759	SRP365980	2022/10/9	1962	6128	1244	4496	35700092
Nicotiana benthamiana	SRX14619767	SRX14619760	SRP365980	2022/10/9	2339	7134	1451	5204	35700092
Oryza sativa	CRX006321	CRX006319	CRA000101	2016/5/10	11599	17049	8264	9977	25483034
Oryza sativa	CRX006323	CRX006322	CRA000101	2016/5/10	9974	18368	8550	12091	25483034
Oryza sativa	CRX017969	CRX017974	CRA000263	2019/12/30	8315	15315	7309	10505	25483034
Oryza sativa	CRX017970	CRX017975	CRA000263	2019/12/30	8457	15949	7582	11134	25483034
Oryza sativa	CRX017971	CRX017976	CRA000263	2019/12/30	7697	18530	6569	11229	25483034
Oryza sativa	CRX017972	CRX017977	CRA000263	2019/12/30	11087	24714	8442	13657	25483034
Oryza sativa	CRX237819	CRX237818	CRA004114	2021/4/27	20718	30752	15276	21122	34633447
Oryza sativa	CRX257287	CRX257286	CRA004530	2021/7/8	26494	35394	18743	21481	25483034
Oryza sativa	CRX257289	CRX257288	CRA004530	2021/7/8	7426	25519	6129	14151	25483034
Oryza sativa	CRX257291	CRX257290	CRA004530	2021/7/8	22477	29009	16353	17636	25483034
Oryza sativa	SRX9973059	SRX9973052	SRP217798	2021/8/13	17179	23209	13506	17665	34294912
Oryza sativa	SRX9973060	SRX9973053	SRP217798	2021/8/13	17317	24808	13851	18146	34294912
Oryza sativa	SRX9973061	SRX9973054	SRP217798	2021/8/13	18383	27308	14621	19287	34294912
Oryza sativa	SRX9973062	SRX9973055	SRP217798	2021/8/13	17966	27464	14284	19410	34294912
Oryza sativa	SRX9973063	SRX9973043	SRP217798	2021/8/13	19808	25964	15001	18815	34294912
Oryza sativa	SRX9973064	SRX9973056	SRP217798	2021/8/13	18570	25717	14348	18937	34294912
Oryza sativa	SRX9973065	SRX9973057	SRP217798	2021/8/13	18292	25208	14662	18667	34294912

Oryza sativa	SRX9973066	SRX9973058	SRP217798	2021/8/13	17824	27634	14118	19485	34294912
Oryza sativa	SRX9973075	SRX9973071	SRP217798	2021/8/13	18901	23342	14302	17751	34294912
Oryza sativa	SRX9973076	SRX9973072	SRP217798	2021/8/13	17425	24387	13617	17999	34294912
Oryza sativa	SRX9973077	SRX9973073	SRP217798	2021/8/13	17667	24887	13709	18321	34294912
Oryza sativa	SRX9973078	SRX9973074	SRP217798	2021/8/13	17944	24984	14033	18502	34294912
Oryza sativa	SRX11013764	SRX11013763	SRP321744	2021/6/1	38604	32754	15762	12857	34167554
Oryza sativa	SRX11013766	SRX11013765	SRP321744	2021/6/1	30342	30718	13006	11835	34167554
Oryza sativa	SRX11013768	SRX11013767	SRP321744	2021/6/1	26630	30393	12458	11999	34167554
Oryza sativa	SRX11013770	SRX11013769	SRP321744	2021/6/1	21926	28567	10249	11438	34167554
Oryza sativa	SRX11013772	SRX11013771	SRP321744	2021/6/1	35403	34672	14369	13799	34167554
Oryza sativa	SRX11013774	SRX11013773	SRP321744	2021/6/1	34209	32649	13895	12634	34167554
Oryza sativa	SRX5705551	SRX5705552	SRP192884	2020/4/22	21204	19841	16992	16889	32341502
Oryza sativa	SRX5705553	SRX5705554	SRP192884	2020/4/22	19614	18480	16023	16126	32341502
Oryza sativa	SRX5705555	SRX5705556	SRP192884	2020/4/22	43195	23648	15815	16471	32341502
Oryza sativa	SRX5705557	SRX5705558	SRP192884	2020/4/22	32953	19925	14557	14171	32341502
Oryza sativa	SRX17687024	SRX17687032	SRP399354	2022/11/3	11233	22073	9998	17728	DOI:10.1016/j.envexpbot.2022.105130
Oryza sativa	SRX17687025	SRX17687033	SRP399354	2022/11/3	11953	23307	10581	18735	DOI:10.1016/j.envexpbot.2022.105130
Oryza sativa	SRX17687028	SRX17687034	SRP399354	2022/11/3	11977	23364	10664	18900	DOI:10.1016/j.envexpbot.2022.

									105130
Oryza sativa	SRX17687029	SRX17687035	SRP399354	2022/11/3	10948	23197	10025	18947	DOI:10.1016/j.envexpbot.2022.105130
Oryza sativa	SRX17687030	SRX17687026	SRP399354	2022/11/3	10956	23214	10169	19199	DOI:10.1016/j.envexpbot.2022.105130
Oryza sativa	SRX17687031	SRX17687027	SRP399354	2022/11/3	12107	24470	11107	20026	DOI:10.1016/j.envexpbot.2022.105130
Oryza sativa	SRX9770033	SRX9768617	SRP300071; SRP300057	2021/8/2	15085	22257	10306	12597	NA
Oryza sativa	SRX9770034	SRX9768618	SRP300071; SRP300057	2021/8/2	16654	26719	10862	14047	NA
Oryza sativa	SRX9770035	SRX9768620	SRP300071; SRP300057	2021/8/2	19344	22619	10396	11534	NA
Oryza sativa	SRX9770036	SRX9768621	SRP300071; SRP300057	2021/8/2	13936	19837	9609	11602	NA
Oryza rufipogon	NA	NA	CRA011747	2023/7/6	18581	21569	11893	15677	NA
Oryza rufipogon	NA	NA	CRA011747	2023/7/6	15508	17015	11217	12663	NA
Paulownia fortunei	SRX13243512	SRX13243511	SRP348213	2021/11/28	16591	23646	10674	14480	NA
Paulownia fortunei	SRX13243514	SRX13243513	SRP348213	2021/11/28	15655	24837	10136	14857	NA
Paulownia fortunei	SRX13243516	SRX13243515	SRP348213	2021/11/28	15861	24640	10172	14618	NA

Paulownia fortunei	SRX13243518	SRX13243517	SRP348213	2021/11/28	15843	23393	10348	14123	NA
Physcomitrium patens	CRX237821	CRX237820	CRA004114	2021/4/27	19115	32448	14884	19957	34633447
Populus trichocarpa	SRX10692441	SRX10692449	SRP316631	2022/5/31	11000	25699	9537	17792	NA
Populus trichocarpa	SRX10692442	SRX10692450	SRP316631	2022/5/31	10153	23355	9122	16653	NA
Populus trichocarpa	SRX10692445	SRX10692451	SRP316631	2022/5/31	9858	23271	8939	16781	NA
Populus trichocarpa	SRX10692446	SRX10692452	SRP316631	2022/5/31	9814	24318	8880	16991	NA
Populus trichocarpa	SRX10692447	SRX10692443	SRP316631	2022/5/31	10353	23697	9226	16958	NA
Populus trichocarpa	SRX10692448	SRX10692444	SRP316631	2022/5/31	9990	22964	9012	16508	NA
Populus trichocarpa	SRX14057364	SRX14057365	SRP358549	2022/2/6	2877	8354	2556	5879	NA
Populus trichocarpa	SRX14057366	SRX14057367	SRP358549	2022/2/6	2312	9246	2110	6475	NA
Populus trichocarpa	SRX14057368	SRX14057369	SRP358549	2022/2/6	3091	8890	2727	6297	NA
Populus trichocarpa	SRX14057370	SRX14057371	SRP358549	2022/2/6	2571	8535	2296	6062	NA
Populus trichocarpa	SRX14057376	SRX14057377	SRP358549	2022/2/6	2642	9349	2369	6622	NA
Populus trichocarpa	SRX14057378	SRX14057379	SRP358549	2022/2/6	2697	9418	2425	6564	NA
Populus trichocarpa	SRX9239577	SRX9239578	SRP241868	2020/10/22	17265	25431	14893	18646	33413586
Phaseolus vulgaris	CRX237805	CRX237804	CRA004114	2021/4/27	18428	24613	12366	17709	34633447
Sorghum bicolor	CRX237809	CRX237808	CRA004114	2021/4/27	21404	27193	14708	18982	34633447
Sorghum bicolor	SRX7909020	SRX7909021	SRP252706	2022/5/3	5672	24760	4773	14520	NA
Sorghum bicolor	SRX7909022	SRX7909023	SRP252706	2022/5/3	5853	21327	4769	13048	NA
Sorghum bicolor	SRX7909024	SRX7909025	SRP252706	2022/5/3	5392	25063	4667	15511	NA
Sorghum bicolor	SRX7909026	SRX7909027	SRP252706	2022/5/3	5118	25908	4202	14414	NA
Sorghum bicolor	SRX7909028	SRX7909029	SRP252706	2022/5/3	1572	27535	1325	14152	NA
Sorghum bicolor	SRX7909030	SRX7909031	SRP252706	2022/5/3	3428	22099	3092	14093	NA
Sorghum bicolor	SRX7909032	SRX7909033	SRP252706	2022/5/3	5898	25663	5038	15740	NA
Sorghum bicolor	SRX7909034	SRX7909035	SRP252706	2022/5/3	7872	24596	6558	15411	NA
Solanum lycopersicum	SRX5260784	SRX5260787	SRP180146	2019/7/21	17159	23463	13209	15071	31387610

Solanum lycopersicum	SRX5260785	SRX5260788	SRP180146	2019/7/21	16126	22643	12684	14828	31387610
Solanum lycopersicum	SRX5260786	SRX5260789	SRP180146	2019/7/21	16917	23690	13089	15246	31387610
Solanum lycopersicum	SRX5260790	SRX5260793	SRP180146	2019/7/21	16329	22729	12825	14791	31387610
Solanum lycopersicum	SRX5260791	SRX5260794	SRP180146	2019/7/21	16938	22066	12994	14681	31387610
Solanum lycopersicum	SRX5260792	SRX5260795	SRP180146	2019/7/21	15552	22434	12341	14441	31387610
Solanum lycopersicum	SRX5260796	SRX5260799	SRP180146	2019/7/21	17351	23714	13228	15756	31387610
Solanum lycopersicum	SRX5260797	SRX5260800	SRP180146	2019/7/21	17034	24153	13077	15488	31387610
Solanum lycopersicum	SRX5260798	SRX5260801	SRP180146	2019/7/21	16426	23327	12982	15771	31387610
Solanum lycopersicum	SRX10311117	SRX10311120	SRP310187	2021/4/13	17844	26050	14032	16935	NA
Solanum lycopersicum	SRX10311118	SRX10311120	SRP310187	2021/4/13	18907	26914	14522	17134	NA
Solanum lycopersicum	SRX10311119	SRX10311120	SRP310187	2021/4/13	17937	26406	14182	17180	NA
Solanum lycopersicum	SRX10311121	SRX10311124	SRP310187	2021/4/13	18987	26710	14237	17083	NA
Solanum lycopersicum	SRX10311122	SRX10311124	SRP310187	2021/4/13	18195	26104	13962	16792	NA
Solanum lycopersicum	SRX10311123	SRX10311124	SRP310187	2021/4/13	18324	26021	14030	16858	NA
Solanum lycopersicum	SRX11230041	SRX11230043	SRP325630	2021/7/3	2846	16076	2368	9172	NA
Solanum lycopersicum	SRX11230042	SRX11230044	SRP325630	2021/7/3	2413	15589	2113	8910	NA
Solanum lycopersicum	SRX11230045	SRX11230047	SRP325630	2021/7/3	2773	16848	2409	9667	NA
Solanum lycopersicum	SRX11230046	SRX11230048	SRP325630	2021/7/3	2773	16876	2394	9794	NA
Solanum lycopersicum	SRX11230049	SRX11230051	SRP325630	2021/7/3	3033	17404	2546	9805	NA
Solanum lycopersicum	SRX11230050	SRX11230052	SRP325630	2021/7/3	3150	17497	2602	9898	NA
Triticum aestivum	CRX237817	CRX237816	CRA004114	2021/4/27	54053	54175	34683	45140	34633447
Triticum aestivum	SRX9933621	SRX9933629	SRP303177	2021/1/29	3395	5266	605	4573	34122371
Triticum aestivum	SRX9933625	SRX9933622	SRP303177	2021/1/29	40798	44263	28266	38927	34122371
Triticum aestivum	SRX9933627	SRX9933623	SRP303177	2021/1/29	39489	45738	28377	38889	34122371
Triticum aestivum	SRX9933628	SRX9933624	SRP303177	2021/1/29	37336	45660	27563	39093	34122371
Triticum aestivum	SRX9933630	SRX9933620	SRP303177	2021/1/29	37984	45626	27640	38814	34122371

Triticum aestivum	SRX9933631	SRX9933626	SRP303177	2021/1/29	36016	47866	26697	43450	34122371
Triticum dicoccoides	CRX237815	CRX237814	CRA004114	2021/4/27	34870	41890	22197	29571	34633447
Zea mays	CRX237811	CRX237810	CRA004114	2021/4/27	26227	28974	15472	21561	34633447
Zea mays	SRX4391354	SRX4391355	SRP153627	2019/6/17	15673	28977	12436	18403	NA
Zea mays	SRX4391356	SRX4391357	SRP153627	2019/6/17	22081	20066	14477	16863	NA
Zea mays	SRX4391359	SRX4391358	SRP153627	2019/6/17	15381	20521	11516	15174	NA
Zea mays	SRX4391361	SRX4391360	SRP153627	2019/6/17	17806	17346	12342	14777	NA
Zea mays	SRX4391363	SRX4391362	SRP153627	2019/6/17	19749	17973	13454	15919	NA
Zea mays	SRX4391365	SRX4391364	SRP153627	2019/6/17	19098	20143	13619	15864	NA
Zea mays	SRX4391367	SRX4391366	SRP153627	2019/6/17	17993	17922	13201	15629	NA
Zea mays	SRX4391369	SRX4391368	SRP153627	2019/6/17	17210	19160	13308	15240	NA
Zea mays	SRX4391371	SRX4391370	SRP153627	2019/6/17	23764	21127	15754	17992	NA
Zea mays	SRX4391373	SRX4391372	SRP153627	2019/6/17	27679	20983	15888	17963	NA
Zea mays	SRX4391375	SRX4391374	SRP153627	2019/6/17	19064	19213	13819	16011	NA
Zea mays	SRX4391377	SRX4391376	SRP153627	2019/6/17	25433	21986	16180	18487	NA
Zea mays	SRX5192958	SRX5192962	SRP175000	2019/9/16	18712	21234	11936	15015	33606877; 31591151
Zea mays	SRX5192959	SRX5192963	SRP175000	2019/9/16	18060	20558	11974	14840	33606877; 31591151
Zea mays	SRX5192960	SRX5192964	SRP175000	2019/9/16	18820	20600	11169	14442	33606877; 31591151
Zea mays	SRX5192961	SRX5192965	SRP175000	2019/9/16	16312	18621	10618	13548	33606877; 31591151
Zea mays	SRX6922056	SRX6922055	SRP223671	2020/5/27	11177	37472	8572	20594	33016611
Zea mays	SRX6922058	SRX6922057	SRP223671	2020/5/27	7734	33632	6690	20039	33016611
Zea mays	SRX6922060	SRX6922059	SRP223671	2020/5/27	15265	40009	10880	21222	33016611

Zea mays	SRX6922062	SRX6922061	SRP223671	2020/5/27	15570	42006	11078	21601	33016611
Zea mays	SRX6922064	SRX6922063	SRP223671	2020/5/27	12177	37589	9210	20914	33016611
Zea mays	SRX6922066	SRX6922065	SRP223671	2020/5/27	10325	35614	8029	20113	33016611
Zea mays	SRX6922069	SRX6922068	SRP223671	2020/5/27	9019	35110	7271	19810	33016611
Zea mays	SRX6922071	SRX6922070	SRP223671	2020/5/27	8112	34321	6548	19590	33016611
Zea mays	SRX6922072	SRX6922067	SRP223671	2020/5/27	10238	36369	7845	20015	33016611
Zea mays	SRX6922074	SRX6922073	SRP223671	2020/5/27	9123	37470	7445	20712	33016611
Zea mays	SRX6922076	SRX6922075	SRP223671	2020/5/27	9501	34995	7636	19923	33016611
Zea mays	SRX6922078	SRX6922077	SRP223671	2020/5/27	7665	32511	6724	19858	33016611
Zea mays	SRX8913958	SRX8913960	SRP276982	2021/4/14	19898	22109	11893	15677	33606877
Zea mays	SRX8913959	SRX8913961	SRP276982	2021/4/14	31026	30821	14432	18653	33606877

Table S2. Data sources and bioinformatics workflow used tools in PRMD.

Name	Links	Ref
	**Data sources**	
Ensembl Plants database	https://plants.ensembl.org/	[1]
Cotton Functional Genomics Database (CottonFGD)	https://cottonfgd.org/	[2]
Genome Database for Rosaceae (GDR)	https://www.rosaceae.org/	[3]
Phytozome	https://phytozome-next.jgi.doe.gov/	[4]
Sequence Read Archive (SRA) database	https://www.ncbi.nlm.nih.gov/sra	
Genome Sequence Archive (GSA) database	https://ngdc.cncb.ac.cn/gsa/	
DirectRMDB	http://www.rnamd.org/directRMDB/	[5]
GWAS Atlas	https://ngdc.cncb.ac.cn/gwas/	[6]
m^6A-Altas2.0	http://rnamd.org/m6a/index.php	[7]
PlantAPAdb	http://www.bmibig.cn/plantAPAdb/	[8]
REPIC	https://repicmod.uchicago.edu/repic/	[9]
G4ATLAS	https://www.g4atlas.org/	[10]
R-loopAtlas	http://bioinfor.kib.ac.cn/R-loopAtlas/l2/browse_obser.html	[11]
RMBaseV2.0	https://rna.sysu.edu.cn/rmbase/	[12]
RASP	http://rasp.zhanglab.net	[13]
PsORF	http://psorf.whu.edu.cn	[14]
POSTAR3	http://111.198.139.65/index.html	[15]
m^5C-Atlas	http://rnamd.org/m5c-atlas/download.html	[16]
AtMAD	http://119.3.41.228/atmad/index.php	[17]
Rice-eQTL	http://riceqtl.ncpgr.cn/home/	
	**Bioinformatics workflow tools**	
Gffread	https://github.com/gpertea/gffread	[18]
clusterProfler package	https://github.com/YuLab-SMU/clusterProfiler	[19]
ReactomePA packages	http://www.bioconductor.org/packages/ReactomePA	[20]
Sratoolkit	https://github.com/ncbi/sra-tools	
Fastp	https://github.com/OpenGene/fastp	[21]
STAR	https://github.com/alexdobin/STAR	[22]
MACS2	https://pypi.python.org/pypi/MACS2	[23]
Exomepeak2	https://github.com/ZW-xjtlu/exomePeak2	
RNASeQC	https://github.com/getzlab/rnaseqc	[24]
Bedtools	https://github.com/arq5x/bedtools2	[25]
OrthoFinder	https://github.com/davidemms/OrthoFinder	[26]
RNAmod	https://bioinformatics.sc.cn/RNAmod	[27]

REFERENCES

1. Howe, K.L., et al., *Ensembl 2021*. Nucleic Acids Res, 2021. **49**(D1): p. D884-D891.
2. Zhu, T., et al., *CottonFGD: an integrated functional genomics database for cotton*. BMC Plant Biol, 2017. **17**(1): p. 101.

3. Jung, S., et al., *15 years of GDR: New data and functionality in the Genome Database for Rosaceae.* Nucleic Acids Res, 2019. **47**(D1): p. D1137-D1145.

4. Goodstein, D.M., et al., *Phytozome: a comparative platform for green plant genomics.* Nucleic Acids Res, 2012. **40**(Database issue): p. D1178-86.

5 Yuxin, Zhang., et al., DirectRMDB: a database of post-transcriptional RNA modifications unveiled from direct RNA sequencing technology. Nucleic Acids Res, 2023.51(D1):P. D106–D116

6 Liu, Xiaonan., et al., GWAS Atlas: an updated knowledgebase integrating more curated associations in plants and animals. Nucleic Acids Res, 2023.51(D1):P. D969–D976

7 Tang, Y., et al., m6A-Atlas: a comprehensive knowledgebase for unraveling the N6-methyladenosine (m6A) epitranscriptome. Nucleic Acids Res, 49, D134-D143.

8 Zhu, Sheng., et al., PlantAPAdb: A Comprehensive Database for Alternative Polyadenylation Sites in Plants. Plant Physiol 2020, 182(1):228-242.

9 Liu, S., et al., REPIC: a database for exploring the N(6)-methyladenosine methylome. Genome Biol 21:100. 10.1186/s13059-020-02012-4.

10 Yu H, Qi Y, Yang B, et al., G4Atlas: a comprehensive transcriptome-wide G-quadruplex database. Nucleic Acids Res, 2023.51(D1):P. D126–D134

11 Li, Kuan., et al., R-loopAtlas: An integrated R-loop resource from 254 plant species sustained by a deep-learning-based tool. Mol Plant 2022 Dec 19;S1674-2052(22)00447-6.

12 Xuan, J., et al., RMBase v2.0: deciphering the map of RNA modifications from epitranscriptome sequencing data. Nucleic Acids Res, 46, D327-D334.

13 Pan, Li., et al., RASP: an atlas of transcriptome-wide RNA secondary structure probing data. Nucleic Acids Research, Volume 49, Issue D1, 8 January 2021, Pages D183–D191

14 Yanjun, Chen, et al., PsORF: a database of small ORFs in plants. Plant Biotechnol J.2020 Nov;18(11):2158-2160.

15 Weihao, Zhao., et al., POSTAR3: an updated platform for exploring post-transcriptional regulation coordinated by RNA-binding proteins. Nucleic Acids Research, 2022, Vol. 50, Database issue D287–D294

16 Jiongming, Ma., et al., m5C-Atlas: a comprehensive database for decoding and annotating the 5-methylcytosine (m5C) epitranscriptome. Nucleic Acids Res. 2022 Jan 7;50(D1):D196-D203

17 Lan, Y., et al., AtMAD: Arabidopsis thaliana multi-omics association database. Nucleic Acids Res. 2021 Jan 8;49(D1):D1445-D1451

18 Pertea, G. and M. Pertea, *GFF Utilities: GffRead and GffCompare.* F1000Res, 2020. **9**.

19 Yu, G., et al., *clusterProfiler: an R package for comparing biological themes among gene clusters.* OMICS, 2012. **16**(5): p. 284-7.

20 Yu, G. and Q.Y. He, *ReactomePA: an R/Bioconductor package for reactome pathway analysis and visualization.* Mol Biosyst, 2016. **12**(2): p. 477-9.

21 Chen, S., et al., *fastp: an ultra-fast all-in-one FASTQ preprocessor.* Bioinformatics, 2018. **34**(17): p. i884-i890.

22 Dobin, A., et al., *STAR: ultrafast universal RNA-seq aligner.* Bioinformatics, 2013. **29**(1): p. 15-21.

23 Zhang, Y., et al., *Model-based analysis of ChIP-Seq (MACS).* Genome Biol, 2008. **9**(9): p. R137.

24 DeLuca, D.S., et al., *RNA-SeQC: RNA-seq metrics for quality control and process optimization.* Bioinformatics, 2012. **28**(11): p. 1530-2.

25 Quinlan, A.R., *BEDTools: The Swiss-Army Tool for Genome Feature Analysis.* Curr Protoc Bioinformatics, 2014. **47**: p. 11 12 1-34.

26 Emms, D.M. and S. Kelly, *OrthoFinder: phylogenetic orthology inference for comparative genomics.* Genome Biol, 2019. **20**(1): p. 238.

27 Liu, Q. and R.I. Gregory, *RNAmod: an integrated system for the annotation of mRNA modifications.* Nucleic Acids Res, 2019. **47**(W1): p. W548-W555.

Table S3. The data sources from previous published research articles.

Data type	Species	Article title	Pubmed id	Ref
m^5C	*Arabidopsis thaliana*	m5C-Atlas: a comprehensive database for decoding and annotating the 5-methylcytosine (m5C) epitranscriptome	34986603	(1)
m^5C	*Arabidopsis thaliana*	5-Methylcytosine RNA Methylation in Arabidopsis Thaliana	28965832	(2)
m^5C	*Arabidopsis thaliana*	Transcriptome-Wide Mapping of RNA 5-Methylcytosine in Arabidopsis mRNAs and Noncoding RNAs	28062751	(3)
m^5C	*Oryza sativa*	OsNSUN2-Mediated 5-Methylcytosine mRNA Modification Enhances Rice Adaptation to High Temperature	32275888	(4)
Pseudouridine	*Arabidopsis thaliana*	Transcriptome-wide analysis of pseudouridylation of mRNA and non-coding RNAs in Arabidopsis	31173101	(5)
eQTL	*Arabidopsis thaliana*	AtMAD: Arabidopsis thaliana multi-omics association database	33219693	(6)
eQTL	*Arabidopsis thaliana*	Large-scale identification of expression quantitative trait loci in Arabidopsis reveals novel candidate regulators of immune responses and other processes	32246811	(7)
eQTL	*Arabidopsis thaliana*	Meta Gene Regulatory Networks in Maize Highlight Functionally Relevant Regulatory Interactions	32184350	(8)
eQTL	*Oryza sativa*	eQTLs play critical roles in regulating gene expression and identifying key regulators in rice	36087348	(9)
eQTL	*Oryza sativa*	Genome-wide expression quantitative trait locus studies facilitate isolation of causal genes controlling panicle structure	32072700	(10)
ac4C	*Arabidopsis thaliana/ Oryza sativa*	Transcriptome-wide profiling of RNA N4-cytidine acetylation in Arabidopsis thaliana and Oryza sativa	37073130	(11)
conservation	*Arabidopsis thaliana*	Regulatory Impact of RNA Secondary Structure across the Arabidopsis Transcriptome[W][OA]	23150631	(12)

REFERENCES

1. Ma, J., Song, B., Wei, Z., Huang, D., Zhang, Y., Su, J., de Magalhaes, J.P., Rigden, D.J., Meng, J. and Chen, K. (2022) m5C-Atlas: a comprehensive database for decoding and annotating the 5-methylcytosine (m5C) epitranscriptome. *Nucleic Acids Res*, **50**, D196-D203.
2. Cui, X., Liang, Z., Shen, L., Zhang, Q., Bao, S., Geng, Y., Zhang, B., Leo, V., Vardy, L.A., Lu, T. *et al.* (2017) 5-Methylcytosine RNA Methylation in Arabidopsis Thaliana. *Mol Plant*, **10**, 1387-1399.
3. David, R., Burgess, A., Parker, B., Li, J., Pulsford, K., Sibbritt, T., Preiss, T. and Searle, I.R. (2017) Transcriptome-Wide Mapping of RNA 5-Methylcytosine in Arabidopsis

mRNAs and Noncoding RNAs. *Plant Cell*, **29**, 445-460.

4. Tang, Y., Gao, C.C., Gao, Y., Yang, Y., Shi, B., Yu, J.L., Lyu, C., Sun, B.F., Wang, H.L., Xu, Y. *et al.* (2020) OsNSUN2-Mediated 5-Methylcytosine mRNA Modification Enhances Rice Adaptation to High Temperature. *Dev Cell*, **53**, 272-286 e277.
5. Sun, L., Xu, Y., Bai, S., Bai, X., Zhu, H., Dong, H., Wang, W., Zhu, X., Hao, F. and Song, C.P. (2019) Transcriptome-wide analysis of pseudouridylation of mRNA and non-coding RNAs in Arabidopsis. *J Exp Bot*, **70**, 5089-5600.
6. Lan, Y., Sun, R., Ouyang, J., Ding, W., Kim, M.J., Wu, J., Li, Y. and Shi, T. (2021) AtMAD: Arabidopsis thaliana multi-omics association database. *Nucleic Acids Res*, **49**, D1445-D1451.
7. Wang, X., Ren, M., Liu, D., Zhang, D., Zhang, C., Lang, Z., Macho, A.P., Zhang, M. and Zhu, J.K. (2020) Large-scale identification of expression quantitative trait loci in Arabidopsis reveals novel candidate regulators of immune responses and other processes. *J Integr Plant Biol*, **62**, 1469-1484.
8. Zhou, P., Li, Z., Magnusson, E., Gomez Cano, F., Crisp, P.A., Noshay, J.M., Grotewold, E., Hirsch, C.N., Briggs, S.P. and Springer, N.M. (2020) Meta Gene Regulatory Networks in Maize Highlight Functionally Relevant Regulatory Interactions. *Plant Cell*, **32**, 1377-1396.
9. Liu, C., Zhu, X., Zhang, J., Shen, M., Chen, K., Fu, X., Ma, L., Liu, X., Zhou, C., Zhou, D.X. *et al.* (2022) eQTLs play critical roles in regulating gene expression and identifying key regulators in rice. *Plant Biotechnol J*, **20**, 2357-2371.
10. Wang, F., Yano, K., Nagamatsu, S., Inari-Ikeda, M., Koketsu, E., Hirano, K., Aya, K. and Matsuoka, M. (2020) Genome-wide expression quantitative trait locus studies facilitate isolation of causal genes controlling panicle structure. *Plant J*, **103**, 266-278.
11. Li, B., Li, D., Cai, L., Zhou, Q., Liu, C., Lin, J., Li, Y., Zhao, X., Li, L., Liu, X. *et al.* (2023) Transcriptome-wide profiling of RNA N(4)-cytidine acetylation in Arabidopsis thaliana and Oryza sativa. *Mol Plant*.
12. Li, F., Zheng, Q., Vandivier, L.E., Willmann, M.R., Chen, Y. and Gregory, B.D. (2012) Regulatory impact of RNA secondary structure across the Arabidopsis transcriptome. *Plant Cell*, **24**, 4346-4359.

Table S4. Other types of RNA modifications.

Methylation type	Organisam	Sample name	Sites count	Annotated genes	Pubmed_ID/ Database
m^{6}A	*Arabidopsis thaliana*	ath_DirectRMDB	10595	5580	36382409
Pseudouridine	*Arabidopsis thaliana*	ath_DirectRMDB	5085	2626	36382409
m^{6}A	*Arabidopsis thaliana*	ath_m6A-Altas2.0	260957	21539	32821938
m^{6}A	*Arabidopsis thaliana*	ath_REPIC	560353	21853	32345346
m^{6}A	*Arabidopsis thaliana*	ath_RMBaseV2.0	20330	4768	29040692
m^{6}A	*Arabidopsis thaliana*	ath_encore_m6A	45720	9762	https://rna.sysu.edu.cn/encore/
2'O-Me	*Arabidopsis thaliana*	ath_encore_Nm	286	11	https://rna.sysu.edu.cn/encore/
Pseudouridine	*Arabidopsis thaliana*	ath_encore_Pseudo	76	7	https://rna.sysu.edu.cn/encore/
m^{5}C	*Arabidopsis thaliana*	ath_m5c_1	684	120	34986603
^{5}C	*Arabidopsis thaliana*	ath_trm4b_4	1780	1490	28965832
m^{5}C	*Arabidopsis thaliana*	ath_Wild_Type	6046	4557	28965832
m^{5}C	*Arabidopsis thaliana*	ath_Root	3468	1225	28062751
m^{5}C	*Arabidopsis thaliana*	ath_Shoot	1020	479	28062751
m^{5}C	*Arabidopsis thaliana*	ath_Silique	1012	516	28062751
Pseudouridine	*Arabidopsis thaliana*	ath_pseudouridine_1	451	337	31173101
ac^{4}C	*Arabidopsis thaliana*	ath_ac4C	3858	2147	37073130
m^{6}A	*Fragaria vesca*	fve_m6A-Altas2.0	43029	9059	32821938
m^{6}A	*Oryza sativa*	osa_m6A-Altas2.0	14917	11237	32821938
m^{5}C	*Oryza sativa*	osa_m5C_1	16991	4736	32275888
ac^{4}C	*Oryza sativa*	osa_ac4C	7508	4185	37073130
m^{1}A	*Phaseolus vulgaris*	pvu_encore_m1A	35	25	https://rna.sysu.edu.cn/encore/
m^{5}C	*Phaseolus vulgaris*	pvu_encore_m5C	19	12	https://rna.sysu.edu.cn/encore/
m^{7}G	*Phaseolus vulgaris*	pvu_encore_m7G	15	7	https://rna.sysu.edu.cn/encore/
2'O-Me	*Phaseolus vulgaris*	pvu_encore_Nm	42	28	https://rna.sysu.edu.cn/encore/

Pseudouridine	*Phaseolus vulgaris*	pvu_encore_Pseudo	78	44	https://rna.sysu.edu.cn/encore/
m^6A	*Populus Trichocarpa*	ptr_DirectRMDB	30690	1158	36382409
Pseudouridine	*Populus Trichocarpa*	ptr_DirectRMDB	10552	745	36382409
m^6A	*Populus trichocarpa*	ptr_m6A-Altas2.0	71926	19944	32821938
m^6A	*Solanum lycopersicum*	sly_m6A-Altas2.0	64852	16205	32821938
m^6A	*Zea Mays*	zma_DirectRMDB	27322	11933	36382409
Pseudouridine	*Zea Mays*	zma_DirectRMDB	20644	6936	36382409
m^6A	*Zea mays*	zma_m6A-Altas2.0	138562	2911	32821938

Table S5. The predicted m⁶A sites for 20 species.

Species	Predicted m^6A-level	Predicted m^6A-sites	Annotated genes
Aegilops tauschii	high	294160	20722
Aegilops tauschii	moderate	353136	24662
Aegilops tauschii	low	364919	25732
Arabidopsis thaliana	high	64558	16446
Arabidopsis thaliana	moderate	73928	18395
Arabidopsis thaliana	low	74551	19009
Brassica rapa	high	37562	19282
Brassica rapa	moderate	42983	21930
Brassica rapa	low	43448	22400
Fragaria vesca	high	108011	20005
Fragaria vesca	moderate	117912	21759
Fragaria vesca	low	115119	21840
Gossypium arboreum	high	41705	21269
Gossypium arboreum	moderate	43999	22537
Gossypium arboreum	low	43204	22493
Gossypium hirsutum	high	134582	41759
Gossypium hirsutum	moderate	148937	45560
Gossypium hirsutum	low	149935	46329
Glycine max	high	132372	55608
Glycine max	moderate	140836	59311
Glycine max	low	143633	60426
Malus domestica	high	49009	23392
Malus domestica	moderate	53452	25350
Malus domestica	low	53237	25661
Nicotiana benthamiana	high	57397	29610
Nicotiana benthamiana	moderate	60183	31282
Nicotiana benthamiana	low	59310	31374
Oryza sativa	high	33559	17371
Oryza sativa	moderate	40813	21021
Oryza sativa	low	43914	22717
Oryza rufipogon	high	30028	11695
Oryza rufipogon	moderate	34600	14037
Oryza rufipogon	low	36242	14987

Paulownia fortunei	high	34145	15920
Paulownia fortunei	moderate	35218	16828
Paulownia fortunei	low	33803	16650
Physcomitrium patens	high	142370	19947
Physcomitrium patens	moderate	167217	21839
Physcomitrium patens	low	167241	22111
Populus trichocarpa	high	100799	44722
Populus trichocarpa	moderate	108416	47935
Populus trichocarpa	low	108110	48032
Phaseolus vulgaris	high	45002	19866
Phaseolus vulgaris	moderate	48263	21329
Phaseolus vulgaris	low	48408	21592
Sorghum bicolor	high	58389	25040
Sorghum bicolor	moderate	70126	29173
Sorghum bicolor	low	73706	30653
Solanum lycopersicum	high	41026	18150
Solanum lycopersicum	moderate	44721	19413
Solanum lycopersicum	low	44698	19657
Triticum aestivum	high	124196	44864
Triticum aestivum	moderate	155971	56605
Triticum aestivum	low	164608	59974
Triticum dicoccoides	high	324140	30877
Triticum dicoccoides	moderate	386152	37688
Triticum dicoccoides	low	400336	39783
Zea mays	high	73210	17483
Zea mays	moderate	91611	22003
Zea mays	low	96070	23384

Table S6. The results of RMplantVar analysis.

Chromosome	Position	Reference	Alter	Transcript ID	Strand	Peak ID	Reference DRACH	Alter DRACH	Reference Score	Alter Score	Gain or Lost
chr1	916013	GCCT	G	Os01t0116300-01	+	SRX5705551_SRX5705552_peak_120	AAACA	TAACA	1	0.995	lost
chr1	916025	CCAT	C	Os01t0116300-01	+	SRX5705551_SRX5705552_peak_120	AAACA	TAACA	1	0.995	lost
chr1	734701	T	TATG	Os01t0113500-01	+	SRX5705551_SRX5705552_peak_80	TAACA	GGACT	0.995	0.946	lost
chr1	2773963	C	G	Os01t0151200-03	+	SRX5705551_SRX5705552_peak_246	AAACT	AGACA	0.978	0.993	gained
chr1	734713	A	ACT	Os01t0113500-01	+	SRX5705551_SRX5705552_peak_80	TAACA	GGACT	0.995	0.946	lost
chr1	734724	G	GAC	Os01t0113500-01	+	SRX5705551_SRX5705552_peak_80	TAACA	GGACT	0.995	0.946	lost
chr1	734725	G	GAGAT	Os01t0113500-01	+	SRX5705551_SRX5705552_peak_80	TAACA	GGACT	0.995	0.946	lost
chr1	734808	G	GGCT	Os01t0113500-01	+	SRX5705551_SRX5705552_peak_80	TAACA	GGACT	0.995	0.946	lost
chr1	734874	G	GGATCA	Os01t0113500-01	+	SRX5705551_SRX5705552_peak_80	TAACA	GGACT	0.995	0.946	lost
chr1	734888	T	TGGC	Os01t0113500-01	+	SRX5705551_SRX5705552_peak_80	TAACA	GGACT	0.995	0.946	lost
chr1	734940	A	ATC	Os01t0113500-01	+	SRX5705551_SRX5705552_peak_80	TAACA	GGACT	0.995	0.946	lost
chr1	736077	C	CTG	Os01t0113500-01	+	SRX5705551_SRX5705552_peak_80	TAACA	GGACT	0.995	0.946	lost
chr1	736125	T	TAA	Os01t0113500-01	+	SRX5705551_SRX5705552_peak_80	TAACA	GGACT	0.995	0.946	lost
chr1	736478	A	ACTC	Os01t0113500-01	+	SRX5705551_SRX5705552_peak_80	TAACA	GGACT	0.995	0.946	lost
chr1	1967183	T	C	Os01t0136400-01	-	SRX5705551_SRX5705552_peak_178	GGACT	GGACC	0.946	0.946	equal

Supplementary Figure legends

Supplementary Figure S1. Characterization of m^{6}A methylomes in 20 plant species. **(A)** Overview of the genome size, gene number, transcriptome-wide m^{6}A methylation ratio and mean m^{6}A methylation ratio among the samples collected for 20 plant species. **(B)** The m^{6}A methylation ratio was negatively correlated with genome size and gene number.

Supplementary Figure S2. Distributions of m^{6}A, m^{5}C and pseudouridine. (A–C) Coverage plot, enriched motif plot and metagene plot of the m^{6}A, m^{5}C and pseudouridine sites.

Supplementary Figure S3. Screenshots of four useful tools in PRMD. RMlevelDiff parameters for analyzing RNA modification levels and differential modifications. RMplantVar was used for detecting potential deleterious variant effects on RNA modifications. RNAmodNet was used for gene co-methylation network analyses and the visualization of the m^{6}A co-modification gene network, while Blast was used for identifying potential RNA modification-related enzymes among the sequences provided by users.

Supplementary Figure S4. Visualization of the IGV data for the association between the agronomic trait-related gene *IPI1* in *Oryza sativa* (Os01g0350900) and its orthologous gene in *Arabidopsis thaliana* (AT3G05545)

Supplementary Figure S5. Orthogroup gene identification and evolution analyses. **(A)** Venn diagram of 1,126 (*Arabidopsis thaliana*), 1,252 (*Oryza sativa*), 1,218 (*Solanum lycopersicum*), 1,608 (*Zea mays*) species-specific orthogroups and 9,715 common orthogroups. **(B)** One-to-one orthologous gene pairs with m^{6}A modifications between *A. thaliana* and *O. sativa*. **(C)** Comparative analysis of the evolution of one-to-one orthologous m^{6}A-modified gene pairs between *A. thaliana* and *O. sativa*. **(D)** Proportion of m^{6}A-modified genes that are associated with agronomic traits.

Supplementary Figure S6. Classification and analyses of co-methylated m^{6}A modules

in *Arabidopsis thaliana*. **(A)** Co-methylated m^6A modules. **(B)** Heatmap presenting the m^6A indices of all co-methylation modules across all *A. thaliana* samples. **(C)** Density distributions of m^6A peaks in different modules across the 5′ UTR, CDS and 3′ UTR. **(D)** Density distributions of the log-transformed lengths of the internal exons with m^6A peaks in different combined co-methylation modules. **(E-H)** In A. thaliana, modules M2 (E), M10 (F), M48 (G) and M55 (H) were associated with panicle size, male sterility/starch content, sucrose content/sugar content and male sterility, respectively.

Supplementary Figure S7. Comparative analysis of wild rice (*Oryza rufipogon*) and two cultivated rice subspecies (*Oryza sativa* ssp. *indica* and *Oryza sativa* ssp. *japonica*). **(A)** Density distributions of m^6A peaks across the 5′ UTR, CDS and 3′ UTR. **(B)** m^6A methylation ratio of all genes in *O. rufipogon*, *O. sativa* ssp. *indica* and *O. sativa* ssp. *japonica*. **(C)** m^6A methylation ratio of singleton genes, gene pairs and gene clusters (NLR gene).
